# Varieties of pictorial vision

**DOI:** 10.1177/20416695241267371

**Published:** 2024-09-02

**Authors:** Jan Koenderink, Andrea van Doorn, Johan Wagemans

**Affiliations:** KU Leuven, Leuven, Belgium; 8125 Utrecht University, Utrecht, The Netherlands; KU Leuven, Leuven, Belgium

**Keywords:** pictorial space, personal differences, heuristics in vision, correlations between judgments

## Abstract

Pictorial awareness is addressed through experimental phenomenology involving over 90 naïve participants. Since one can’t look at the “same” picture twice the study uses one-shot trials. The participant’s fascination for the duration of a session is held through the artistic principle of theme and variation. Six variations focus on the theme of pictorial geometry, both two-dimensional and three-dimensional. Major findings are:

Idiosyncratic deviations from veridical are huge as compared to common textbook “effects.”

Observers wield arbitrary heuristics for tasks that are “formally related.” The assumption of a common formal framework is apparently unsound. The notion of “inverse optics” is misleading.

A fair fraction of the population appears to lack monocular stereopsis as intuitive awareness. It suggests an as-yet unrecognized, but perhaps common variety of aphantasia.

## Introduction

To face the issue of “pictorial space,” one needs to carefully distinguish ontological levels. In discussing a painting executed *en plein air* one should at least recognize:

- The scene in front of the picture crafter. It is described in terms of physics, say particles, radiations, and so forth. That would satisfy a blind scientist, it is “all there is.”
- The topic in the mind of the painter. It is described in terms of artistic meanings and values, implicit in the artist’s imagination. It is a realm that cannot be exhausted, although it would be meaningless to the exact sciences.
- The picture (paintings, photographs, etc.) is a flat surface covered with pigments. Pictures are physical objects. They can be described in terms of arrays of numbers (“pixels”). That would satisfy a blind scientist, it is “all there is.”
- The image in the mind of an observer when contemplating the picture. The relevant aspect of the mind is “visual awareness,” that is actuality. To the sentient mind, actuality is all there is. Images are intuitions, instead of reflective, conceptual thoughts.^1^ They “happen” to one, one doesn’t “do” them. Images in this sense do not exist for the exact sciences.

There need be no scene,^2^ but the topic, picture, and image are invariably involved. This is understood by philosophically inclined artists. We concentrate on instances before the conceptual turn of the early 20th century.

Max Liebermann (1922) (German painter, 1847–1935) explains that the artist always paints from the imagination, even when working *en plein air*. Put a dozen artists in front of a scene and end up with a dozen different renderings. Photographic recording is something entirely different.

Maurice Denis (1890) (French painter, 1870–1943) stresses that a painting is a physical object, much like a tea tray. It is not more than that (Manzotti, 2021) if you are unable to look into it, as is apparently the case with cats and dogs. Denis was a rare visionary, who understood that whereas images are magical, pictures are common physical objects.

Adolf Hildebrand (1893) (German sculptor, 1847–1921) explains the image in the mind of an observer as the *Fernbild*, an iconic, Gestalt-like object.^3^ He describes many of its properties. His is the most relevant phenomenological account we possess today (well over a century later!).

There are iconic objects in the absence of pictures. Think of a painter’s imagery of the scene, or of counterfactual imaginary scenes as Heaven or Hell. Iconicity is a mental, not a physical property.^4^

We are interested in iconicity.^5^ Depicting is a one-way communication channel without a shared language. The communication is “expressive” and “affective,” grounded in common optical and life experience. It is based on signs^6^ that are intentionally used by the picture crafter and “felt” by the picture viewer. Hildebrand explains why pictures may evoke images that feel “realer than real.”

Signs are also found in nature Uexküll (1921, 1934, 1936), as when one sees faces in the clouds. This example shows that such signs are not part of physical nature, but are structures promoted to sign status by the sentience of an observer. In vision research one often speaks of “cues.” Cues also exist only in relation to observers. This is one likely cause of interobserver differences.

Pictorial vision as a communication channel appears closed to animals other than man.^7^ It probably is a cultural achievement in the genus *Homo*. It involves a level of as if (Vaihinger, 1911) between sentience and sapience. This is where humor and art live (Koenderink, 2019c).^8^

“Pictorial Perception” refers to the presentation of imagery in visual awareness in the act of picture viewing. That is iconic contemplation. Pictures yield to objective descriptions. Imagery is probed by methods of experimental phenomenology (Albertazzi, 2013, 2021). The study of pictorial perception aims at a formal description of relations between pictures and images. It is “theoretical phenomenology.”

Such descriptions will be very unlike theories in physics. There is no deterministic causation. One deals with uncharted differences between individual *Hominin* and the fragmented and often inconsistent competences found with all animals—including us (Bendaña & Mandelbaum, 2021).

A biological attitude as pioneered by Jacob von Uexküll (1921, 1934, 1936) (German biologist, 1864–1944) understands perception as a process of semiosis. The consequences have hardly been squarely faced. The early Gestalt movement came closest. Bishop Berkeley (1709)) (Irish philosopher, 1685–1753) has already many of the pertinent notions.

From this perspective, there are likely to be major differences among groups of *Homo sapiens*. Human beliefs, judgments, and actions are not necessarily consistent, but more likely to be fragmented according to setting and current goal (Bendaña & Mandelbaum, 2021; Koenderink, 2019b, 2019c).

Picture crafting and viewing—the “pictorial arts”—open up a fruitful field of endeavor. We attempt a concerted foray into the area.

### Modus Operandi

The genus *Homo* is far from homogeneous. Human minds are fragmented and inconsistent (Koenderink, 2015a). Hence the necessity to increase both the number of observers and the number of operationalizations of related topics. It is not obvious how visual awareness can be parcellated in qualities, objects, relations, and so on. The present understanding is flimsy and at best fragmented in narrow foci (Koenderink et al., 2011; Koenderink, 2015a).

This suggests an unmanageable task. We focus on a pragmatic major hurdle, that is, the time limit. Rather than an irrelevancy, it is a key issue. You can only see a picture once, for the next time the image will be different. So repeats are out. Deploying many observers and many tasks implies that the tasks should be simple and fast to perform, yet yield a discriminating datum. This bottleneck hinders the collection of pertinent data. Humble as the choice seems, it is relevant.

We build on decades of experience with a wide range of related issues (Koenderink et al., 2010a, 2010b, 2011; Pont et al., 2012; Koenderink et al., 2012, 2016), so we are well aware of the unmanageable size of the task. We present a minor, initial step, intended to indicate a possible way to progress.

The design of short, yet discriminative experiments is hard. Moreover, it is not something all workers in the field will appreciate. Conventional approaches tend to involve long exposures and many repeats. Yet—we feel—it is the way to proceed.

#### Choice of Topics

Consider some pertinent facts. Each participant should do one session of less than an hour. A session should be made up of perhaps half a dozen short tasks. A few minutes should do. Repeats are to be avoided, since (echoing Heraclitus) one cannot look at the “same picture” twice. A task should consist of one or more trials of a few seconds each. Trials should be easy to do and feel “natural.” It would be best for stimuli to look interesting and manipulations to be fun. Boredom should be avoided at all costs. One way to implement that is to compose the task on the *principle of repetition with variation*.^9^

An ideal might be something like half a dozen tasks^10^ taking no more than a few minutes each. Any task should yield a useful (i.e., characteristic) datum. We pick half a dozen, partly because of conceptual relations, partly serendipitously:

- Visual field extent: The binocular field of view (FOV) subtends about a half-space. It is an object from geometrical optics (physics). The visual field is a spatial aspect (its “size”) of visual awareness. It is idiosyncratic and may differ from the FOV extent by a large factor (Koenderink et al., 2010b). A priori, participants with very different visual field extents are likely to differ in other respects.
- Box-shape in one-point perspective: One-point perspective box spaces were even used before the advent of formal linear perspective. The front and back faces of the box appear as concentric squares. Depending on their size ratio, the box appears as a cuboid ranging from a shallow slab to a deep corridor (Figure 7). For a certain ratio, the rendering will appear box-like, or “cubical.” This ratio is largely idiosyncratic and largely independent of the optical (physical) factors (Pont et al., 2012).
- Spatial attitude in pictorial space: Linear perspective yields major deformations. One is the misrepresentation of spatial attitude, say the “pose” of an object. Artists have “corrected” for that from the renaissance on (Pirenne, 1970). Naive viewers commit huge errors (up to a hundred degrees) in estimating the “correct” spatial attitude in pictures (Koenderink et al., 2010a).
- Pictorial depth: The awareness of the picture when *looking AT* it is that of a flat (two-dimensional [2D]) surface. Many viewers experience “pictorial depth” when looking INTO a picture. Their image is “three-dimensional (3D),” it has “depth.” A fair fraction of viewers appears to lack the depth-quality (Koenderink et al., 2011). They know how to interpret 3D spatial configurations in pictures, but they rely on cognitive judgments, rather than optical intuition. This has led to the notion of a majority of scientists that stereopsis is the causal consequence of binocular disparity.^11^ The likely inhomogeneous composition of the generic population renders this task a useful target.
- “Presence” in pictorial scenes: The feeling of “presence” is often described as “I could enter the picture,” or “I feel like I’m in the scene.” Artists know that the feeling of presence can be heightened through selective scaling of relevant parts (Pepperell & Haertel, 2014). The size and slope of pictorial mountains need to be increased in depiction in order to avoid them looking like mole hills. It is not known how viewers differ in this respect, so it seems like a good idea to sample that.
- Aspect ratio of pictures: The aspect ratio of a (rectangular say) picture is a 2D, geometrical parameter. There exist well-known preferences for limited types of display, such as cinema, tv, postcards, photography, and so on. Moreover, the pictorial content and the aspect ratio interact. The art of composition recognizes the essential role of the frame.^12^

There is no such thing as a “best” selection here. Numerous additional topics are feasible. Our understanding is limited. Each topic can be subdivided into numerous subtopics that may well lead to surprising findings. This study is necessarily an initial foray.

#### Implementation

It makes no difference to the results what hardware and/or software is used. Just for the record:

- Programs were implemented in Processing 4 on an Apple powerbook. Processing is a form of Java,^13^ thus the code is largely platform-independent. It runs without change under Windows on pcs.
- Programs were run on a desktop PC under Windows, except for the visual field task, which was run on a small Apple PowerBook.
- Analysis was done in Mathematica v12.0.0.0.^14^ One needs exploratory data analysis, relying on graphics and varieties of nonparametric methods. Such methods rely on intuitions honed through experience.

#### Presentation

Except for the visual field task, we used a large monitor (screen size 885×500mm) in a dimly lit room. Viewing distance was either 25 cm (FOV 121∘×90∘ , tasks II and III or 85 cm (FOV 55∘×33∘ , tasks IV, V and VI).

Viewing was binocular and informal except for viewing distance and (for tasks iii, see below) a fixation mark. Although binocular vision is not ideal,^15^ most observers feel more confident that way. It renders the task less artificial to them, so one has little choice.

For tasks involving “pictorial vision,” the screen showed a roughly finished plaster wall with a framed picture superimposed. The frame isolates the picture from the wall and imposes a natural frontal plane.

Different from virtual reality, pictures are man-made objects presented in an environment congenial to viewing them. Thus, simultaneous vision of a picture and its environment is desirable. We aim at a set-up resembling viewing pictures on a gallery wall (Koenderink et al., 2011; Koenderink, 2011; Koenderink et al., 2016; Koenderink, 2019b, 2019c), rather than something approaching “virtual reality.” Unfortunately, we cannot do such experiments in a real gallery setting because we need to implement parametric control.

**Figure 1.**
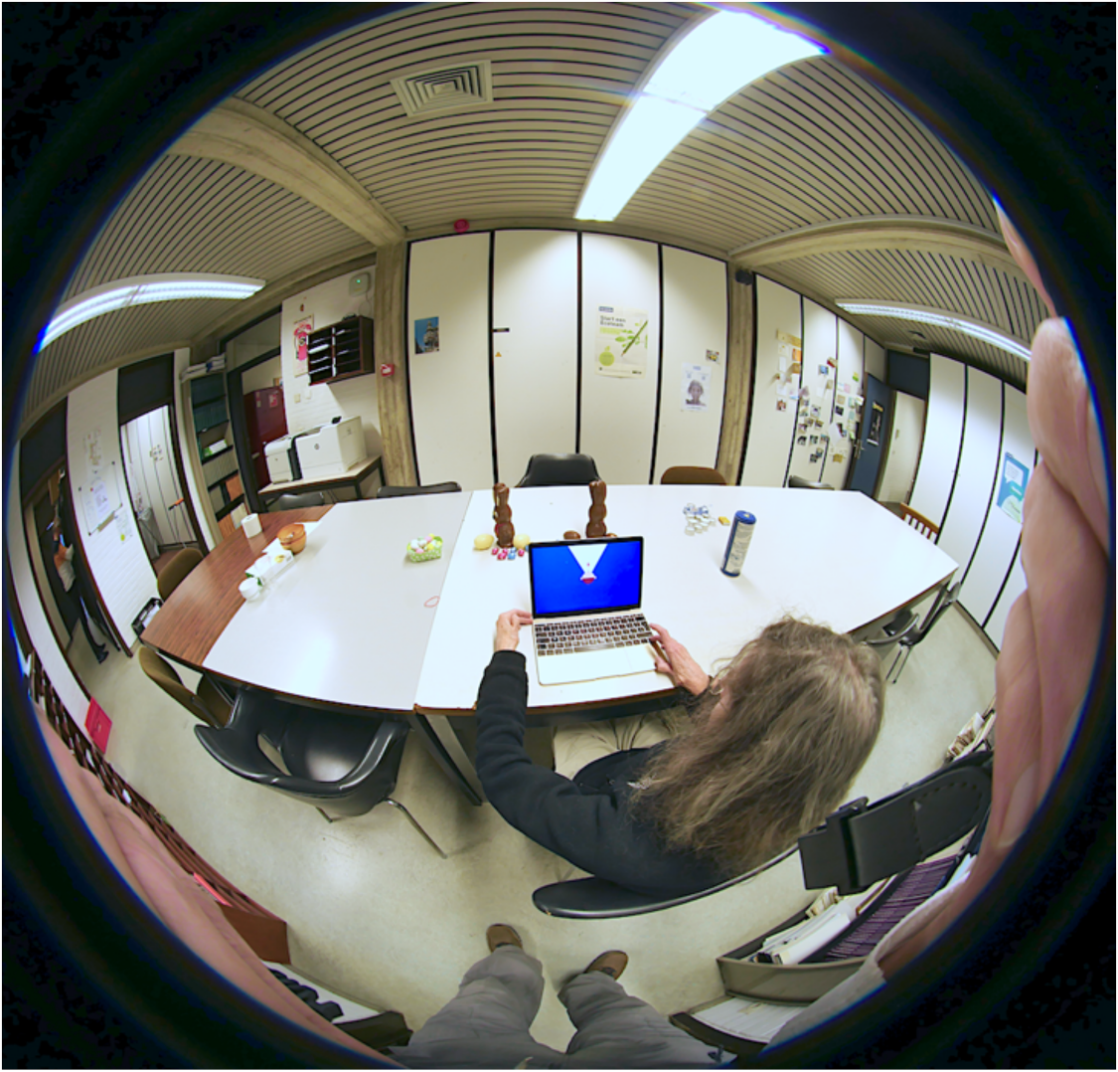
Actor (representing a participant) in the setup. Note the screen with visual field size indicator.

For the visual field task (task I), we used a small, handheld laptop. This task was done in a large open room, in order to ensure that the participant will have a full, detailed FOV (Figure 1). A location in an outside open space is preferable, but was not practical. Viewing was informal. Head movements were discouraged, but eye movements were permitted.

#### Instructions

We use a short verbal introduction in discussion with the participant. This ascertains that the task is understood. It aids in preparing the mind set of the observer. The task starts with a short formal instruction, using both text and illustrations.^16^ The first trial automatically starts after the participant has read the final page of the formal instruction.

#### Participants

Participants were volunteers from post-docs, PhD-students, and technical and administrative staff of the KU Leuven. Apart from Belgian citizens, there were many participants from various Western and Chinese roots. Participants were not involved in the study and had no prior knowledge of its goals and methods. A minority had some familiarity with various strands of vision research. Most were not familiar with the art used as stimuli.

This is a group of naïve observers that—most having opted for academic study—is used to rely on judgments in reflective thought rather than visual intuition. In the verbal instruction sessions, we attempt to convince the participants to respond “on the guts.” Many consider that difficult. From the interactions, we glean that it is not likely to succeed in all cases. Unfortunately, such impressions cannot be used in the formal analysis. This has to be kept in mind when assessing the results.

There are 93 participants in total, about three-quarters female. The age range was unlimited, but largely confined to people in their 20s. All wore their normal correction if necessary and had normal vision by informal criteria and self-reporting.

#### Data Collection

Tasks were performed in random sequence, except that the visual field extent was—for practical reasons—always scheduled as either first or last. For each subtask, the trials were in random sequence.

Each trial is only considered concluded if there has been a formal minimum of interactions.^17^ After the conclusion of a trial, the next one is immediately initiated. We record response times from the initiation of a trial to its conclusion, discarding inter-trial delays.

Response times are in no way reaction times. However, some overall measures such as the median can be permitted as meaningful data.

## Individual Tasks

We use a common format in reporting the tasks:

Concepts: Describes the task and introduces the necessary formal issues.
Method: Describes matters specific to the given task.
Result: Presents a descriptive statistical analysis of the results.
Epilog: Discusses specific issues and possible far-reaching conclusions on the basis of the specific task.

In a later section, we consider relations (if any) between tasks.

### Visual Field Extent

#### Concepts

It is crucial to distinguish between the:

Field of View (FOV): The fov is a concept from geometrical optics. It describes the pencil of rays that is accepted by an optical system. It has nothing to do with vision as such. Even a photographic camera has an fov, so has a blind person with an intact eye (*Seelenblindheit*; (Lissauer, 1890)). The extent of the human fov for a single eye is limited by nose, brows, and cheekbones. We consider the horizontal extent of the binocular FOV. Anatomically it may be as wide as 210∘ . A practical estimate is the half-field in front of the head, thus 180∘ .
Visual Field (VF): The vf is an aspect of visual awareness, anything in view right now. Thus a camera does not have a vf because it is not aware. The extent of the vf cannot be expressed in geometrical/physical terms. We collect subjective estimates.

There is no reason why the width of the vf should be causally related to the width of the fov. Although both measures are denoted “width” (only a word), they are ontologically distinct.

From a historical perspective, we have Kepler’s (Lindberg, 1976) remark that the half-space in front of him seemed to be situated “in front” and Helmholtz’s (Helmholtz, 1867) remark that the width of the visual field seemed more like a right angle to him. Both were remarkable observers who spontaneously noticed a—to them—surprising discrepancy. There are few observers like that—none of our participants reported to have ever experienced such a spontaneous insight. But most had no difficulty in indicating the apparent extent of their visual field, nor did they conceive of the task as strange or impossible.

#### Method

The instructions involve an explanation of the difference between the fov and the vf. We mention the usual data on the fov and the empirical fact that estimates of the extent of the vf vary largely over the population. The participants are told that there is no “right answer” and that any indication of the vf is per definition “correct.” We also mention that persons with very different vf extents cannot be distinguished by either anatomy, physiology. or psychophysics. Neither very large nor very small values are either incorrect or worrisome.

**Figure 2.**
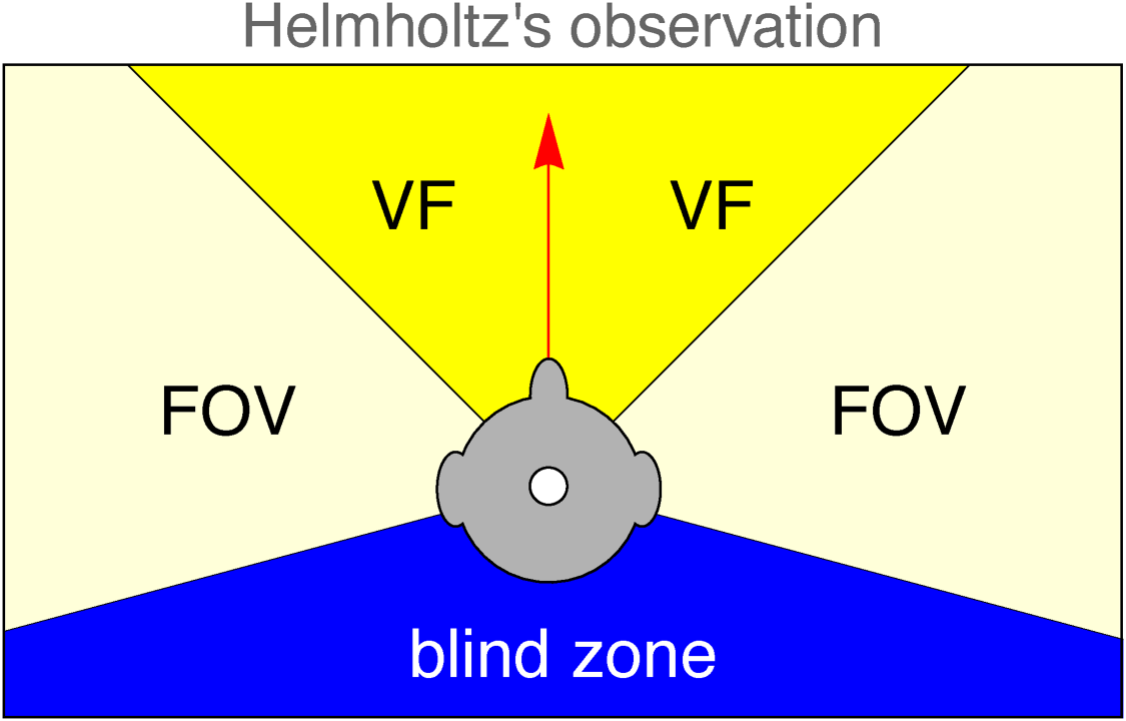
This illustrates the remarkable discrepancy spontaneously noted by Helmholtz between the extent of the fov (about a half-space) and the vf (“about a right-angle”). A similar display was used by the participants to indicate the width of their vf.

Then the participant is asked to indicate the extent of the vf on a laptop display (similar to Figure 2). This is done in a large room with an ample optical structure in all directions (see Figure 1).

The angle indicated by the participant is interpreted as an operationally defined “extent of the visual field.” Such empirical angles are naturally expressed in degrees. Of course, “subjective degrees” are ontologically distinct from “objective degrees.” However, formal geometry takes no account of ontology.

#### Results

**Figure 3.**
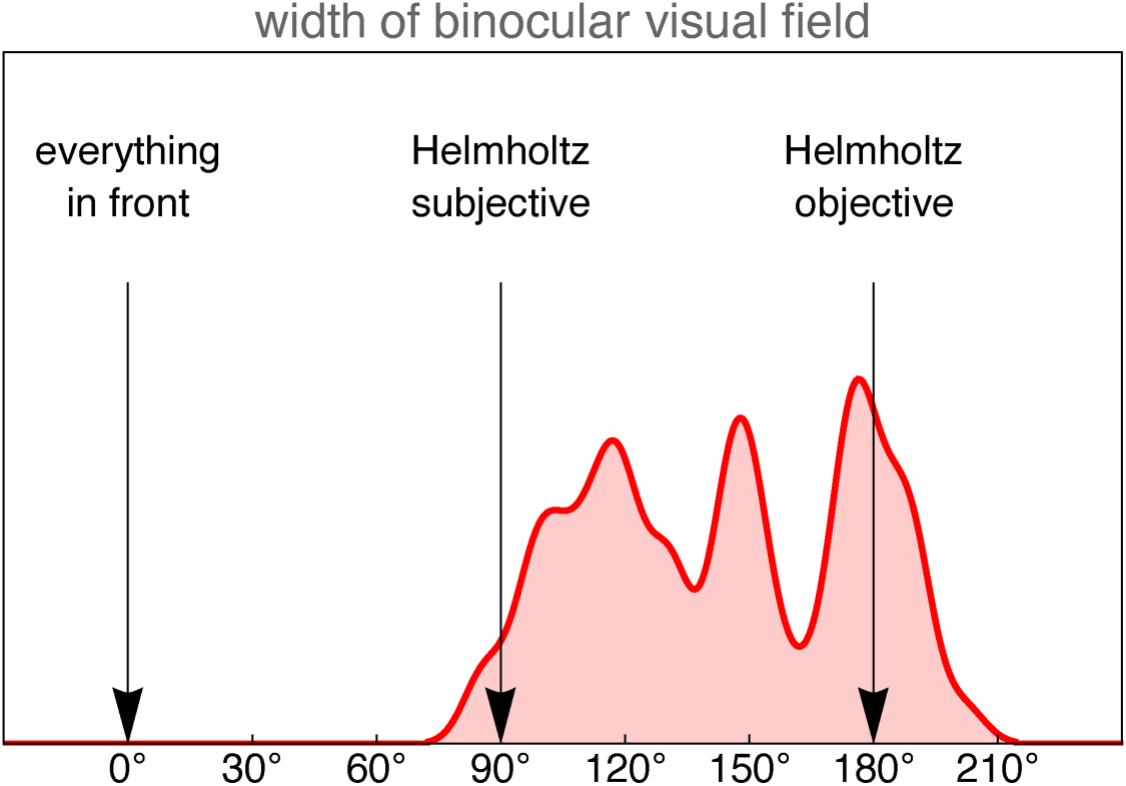
The (smoothed) empirical distribution of the vf size estimates of the group of naive participants.

We find a roughly uniform distribution (Figure 3) ranging from 86∘ to 202∘ (not rejected at the 5% level based on the Cramér–von Mises test).

#### Epilog

In an earlier, far more sophisticated but elaborate, study (Koenderink et al., 2010b) we found an even wider range. Especially very narrow vfs were rare. In this study, they could have been missed due to the limited number of participants.

From post-trial debriefing sessions, we know that some participants “see everything in front of them” (suggesting a very narrow—down to 0∘ —vf), whereas others “feel immersed in the scene” (suggesting a very broad—up to 360∘ —vf). Their convictions appear fragmented and often inconsistent when probed with additional questions, such as “can you see what is behind your back,” and so forth.

The setting and the task no doubt make a difference. Some people reason it out (we told them that the fov is about a half-space, which surprised some participants), others asked “what do you want me to indicate?” or, especially, “did I do it right?”

The latter remark indicates a lack of differentiation between the mental and the physical world.^18^ Such persons are reluctant to perform the Husserlean “bracketing” or “epoché” (Husserl, 1931).

Note that it makes no sense to repeat a trial several times. That would require major temporal gaps (days, weeks) in order to render the trials mutually independent. Thus, we have no such a thing as intraobserver variability. Therefore, we need a large group of participants. However, it is a priori likely that there will be major interobserver differences.

The upshot is that one cannot assume that all estimates address the same mental object. However, our methodology does not permit us to differentiate. Such variety is expected and part of the design of the study. A tentative conclusion is that the width of the vf is idiosyncratic and may well be significantly less or more than a half-space.

This corroborates the remarkable observations by Kepler (Lindberg, 1976) and Helmholtz (1867).

### Cubes in One-Point Perspective

#### Concepts

It is important to distinguish between the following concepts:

Cubes: A cube is one of the classical regular polyhedra. It has six square faces, 12 edges, and eight vertices such that at any vertex three faces (or alsothree edges) meet. It is often defined in terms of formal symmetries. One may also regard it as the convex hull of the vertices with Cartesian coordinates {±1,±1,±1} .
Pictures of cubes: Pictures of cubes are 2D Schlegel diagrams (Schlegel, 1883). We consider one-point linear perspective renderings of wireframe cubes (Alberti, 1435; Cole, 1921; Dunn, 1995; Hamm, 1972).
Images evoked by pictures of cubes: Images evoked by pictures of cubes often fail to “look like a cube.” In fact, only a small subset of all (perfectly veridical) linear perspective renderings of a cube look like a cube at all. Many vision scientists consider this strange, after all shouldn’t a “veridical” rendering settle the matter?

#### Method

**Figure 4.**
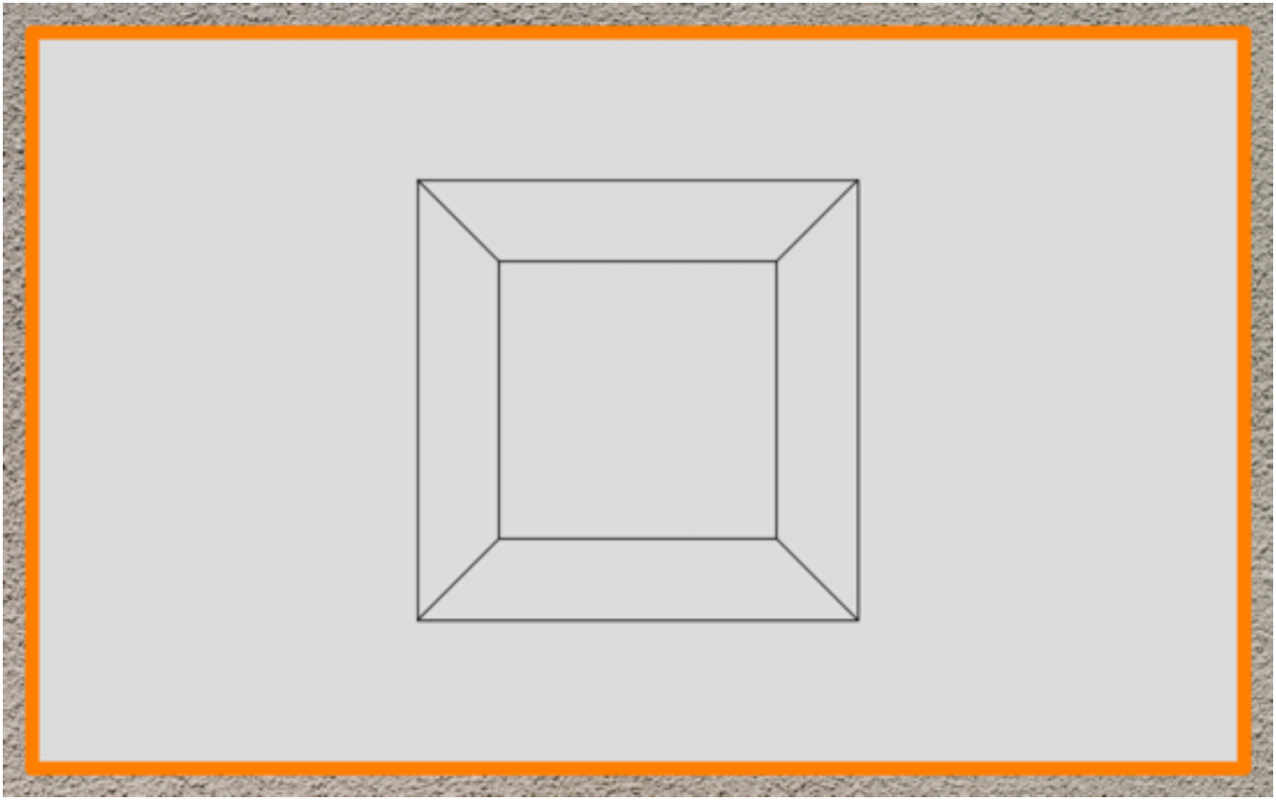
A wire frame cube in one-point perspective. Note that it is presented as a picture with a well-defined picture plane. The participant uses the arrow keys to vary the ratio of the projected front and back edges between very wide limits. This stimulus is presented in a large range of sizes.

**Figure 5.**
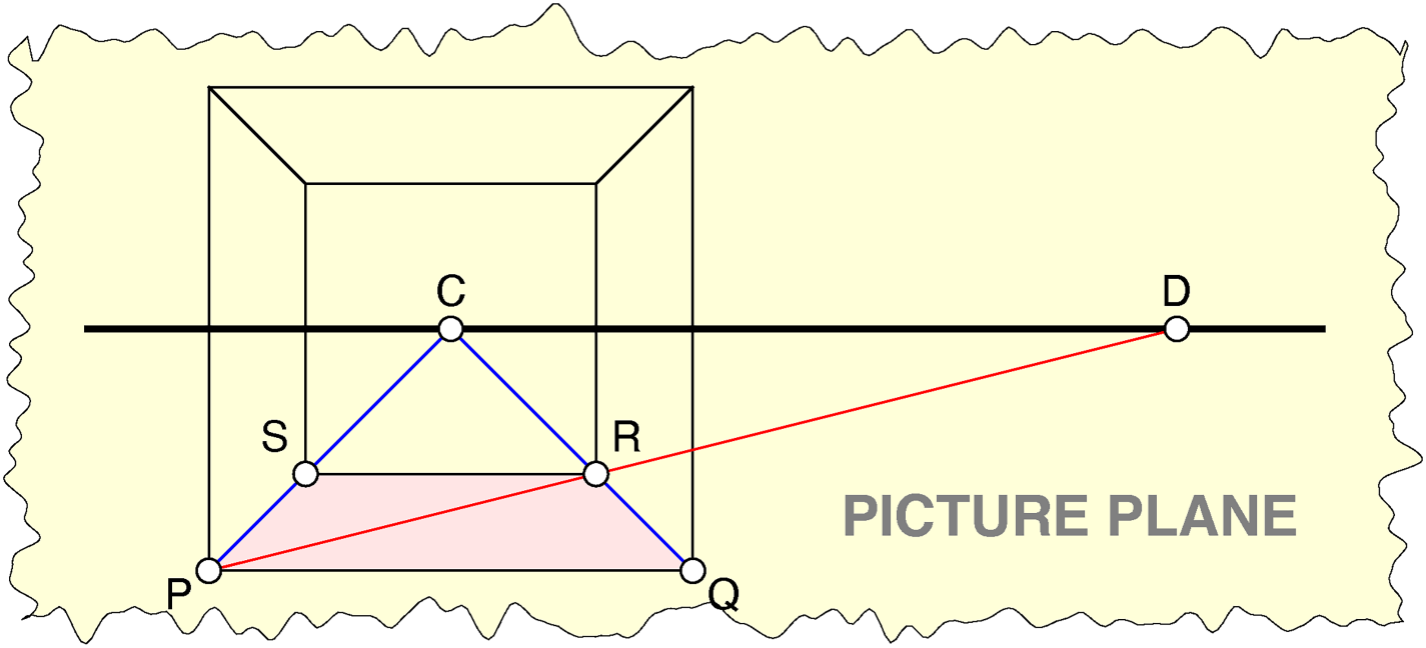
The one-point perspective projection of a cube. Principal vanishing point C, distance point D, ground face PQRS. Note the collinear triples PSC, QRC, and PRD.

**Figure 6.**
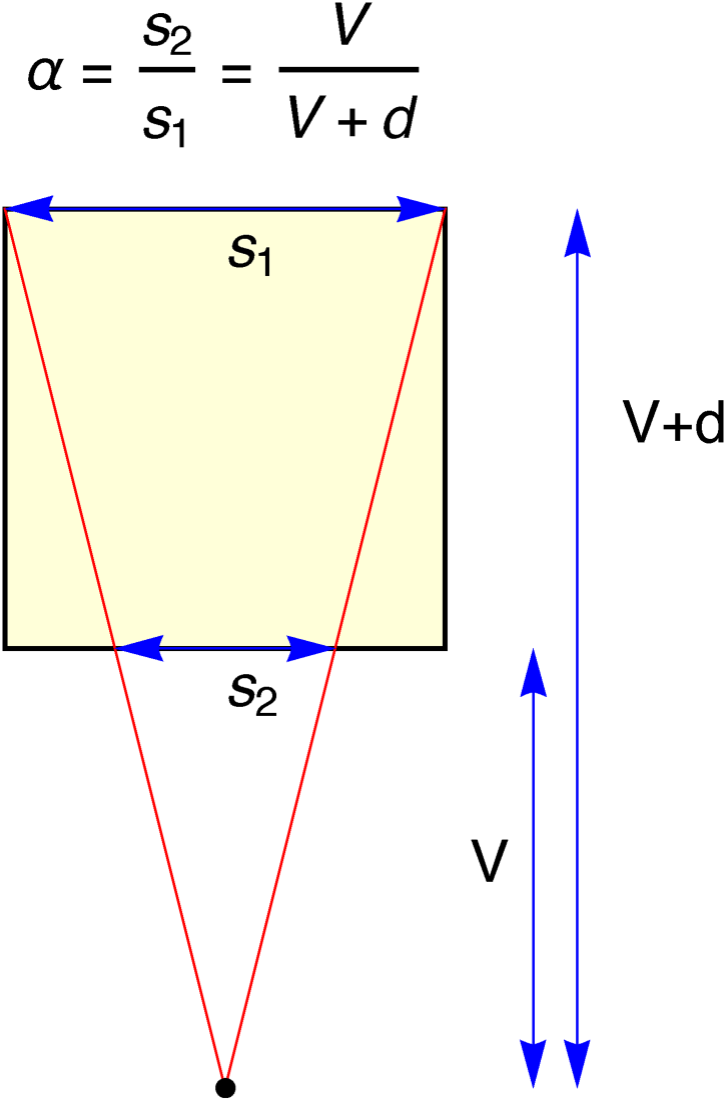
The definition of the parameter α∈(0,1) . This is a ground-plan of Figure 5.

We present cubes in a one-point perspective (Figure 4, (Cole, 1921)). From the basic geometry (see Figure 5) you easily derive the “correct” viewing distance. It depends upon the size of the cube (edge length d) and the perspective (the ratio α , see Figure 6). In the experiment. the actual viewing distance V is fixed, thus—usually—“incorrect.” We used a wide range of edge lengths d.

Participants have real-time control of a one-point perspective of a cube (the parameter α ). In this one-parameter subset of the cube, most renderings look like pictures of shallow slabs, or of pictures of deep corridors.

The task of the observer is to set the parameter α such that the picture “looks like” a regular cube and not like a corridor or a slab.

Viewing distance was 25 cm, yielding a FOV 121∘×90∘ .

##### Psycho-physical correspondence

Figure 5 shows the familiar construction of a one-point perspective rendering of a cube. Point C is the “principal vanishing point,” whereas point D is the “distance point.” Thus, the distance CD in the picture plane equals the “correct” viewing distance.

The frontal face appears as a square. The back face, which is farther away, appears as a smaller, concentric square. In order to construct it one draws the diagonal PR, where point R is found as the intersection of the lines CQ and PD.

We define a convenient shape-parameter α as shown in the diagram shown in Figure 6. The diagram shows the geometry on the “ground plan.” The parameter α is simply the ratio of the front and back edges in the picture (Figure 6). It can also be expressed in terms of the (correct) viewing distance measured in terms of the edge length (thus V/d ).

**Figure 7.**
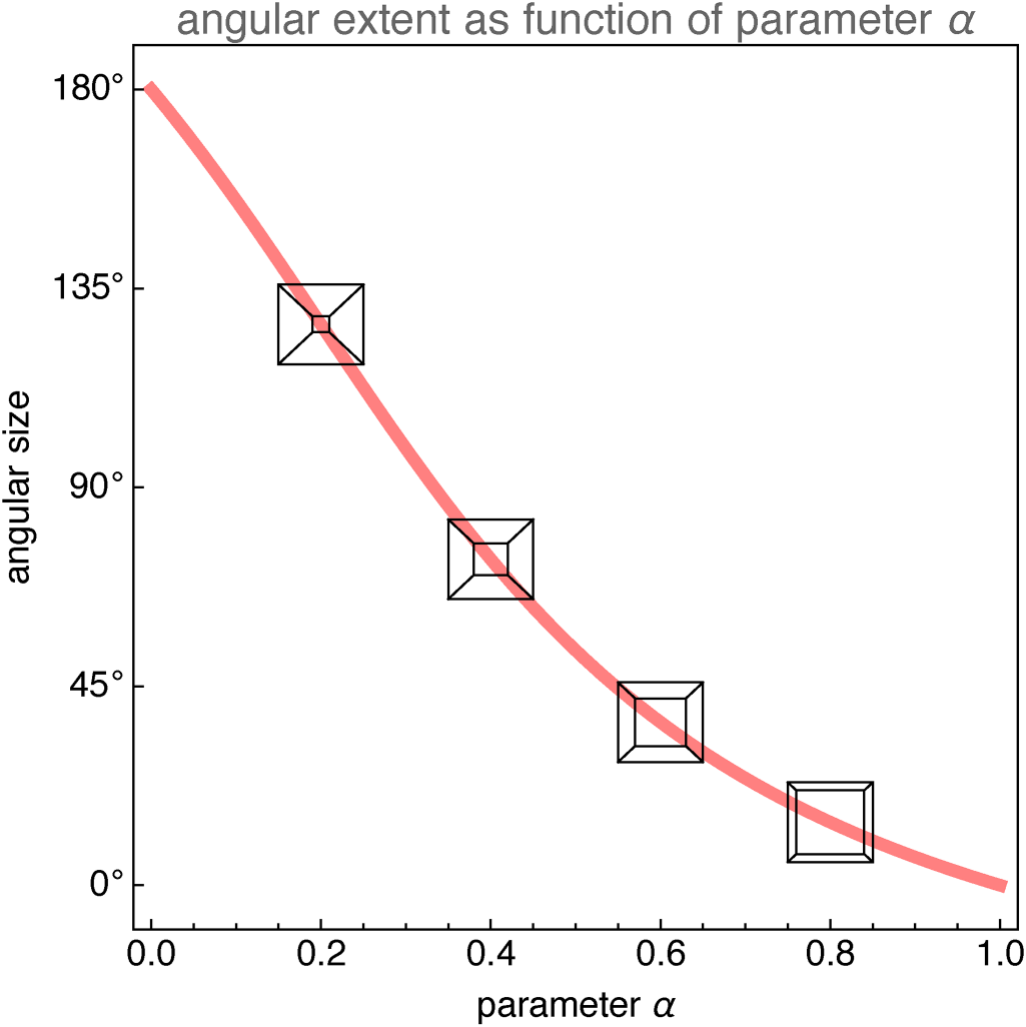
The habitus of the picture of the cube in its dependence on the parameter α . At α=0, the eye is in the front face, thus the visual extent 180∘ . At α=1.0 the eye is at infinity, thus the visual extent 0∘ .

Figure 7 suggests how the habitus^19^ depends upon the shape-parameter. Note that small values of α tend to look like deep corridors, whereas large values tend to look like shallow slabs. Somewhere in the region near α≈0.5 does one (the authors at least) find renderings that suggest a cube.

#### Results

**Figure 8.**
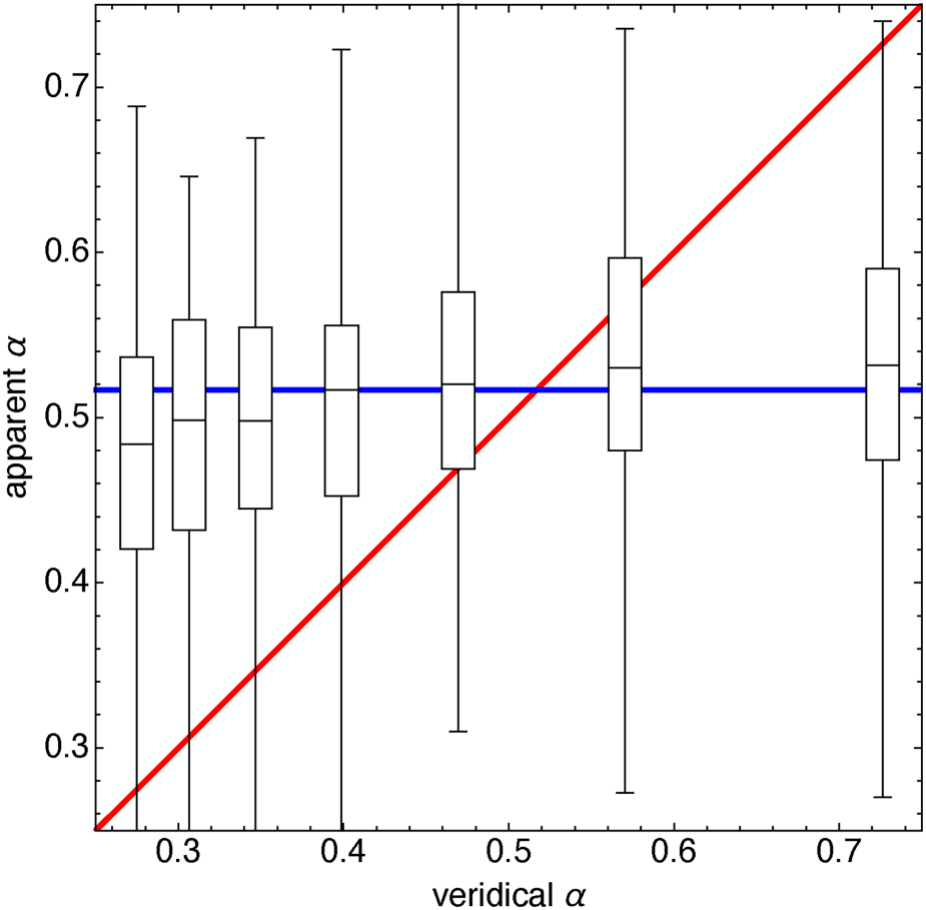
Range and quartiles over all settings of all participants. The constant value (blue) fits the data better than the veridical value (red).

Figure 8 shows ranges and quartiles of all settings. The red line would imply a veridical (“correct”) response. Note that the actual medians are perhaps better described by the blue line, which greatly deviates from veridicality.

**Figure 9.**
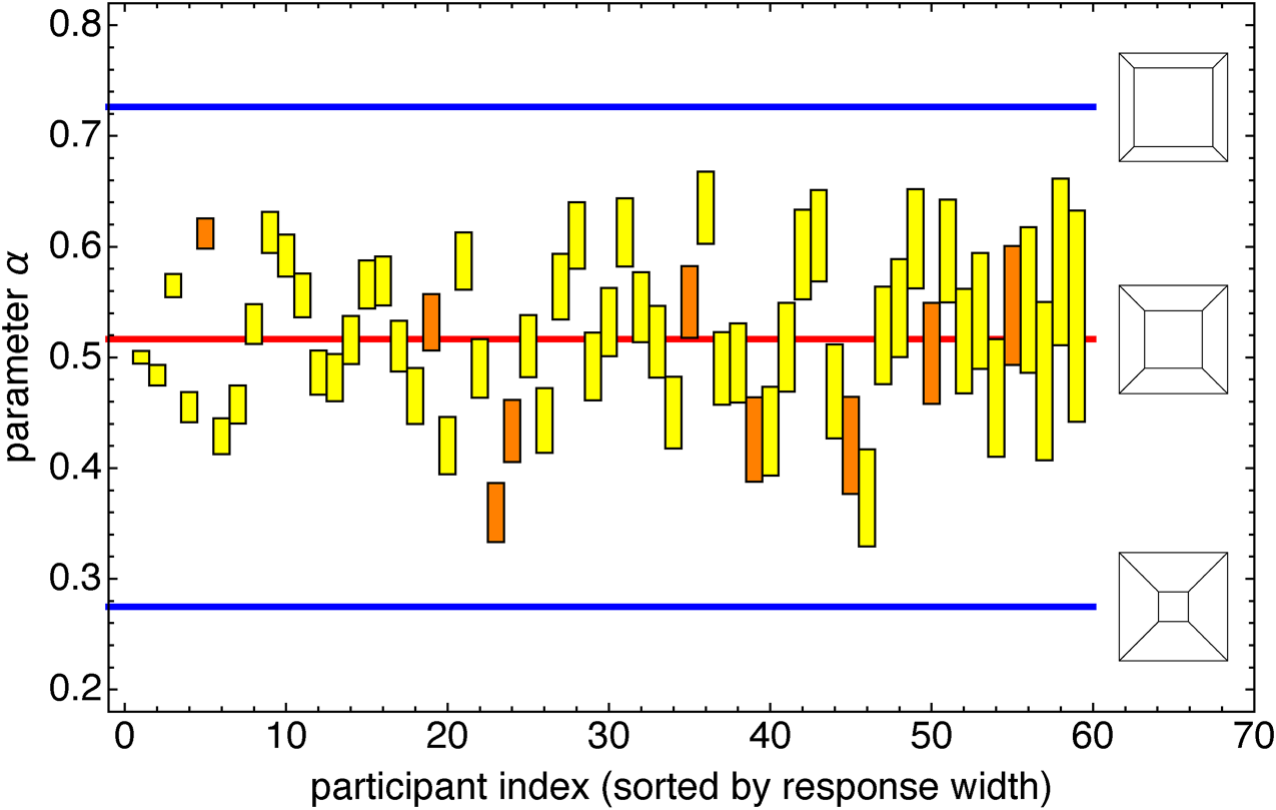
Individual interquartile ranges of all participants. The blue lines indicate the extreme values used in the experiment. The red line suggests the overall median. The orange ranges correlate with the veridical values at the 5% level, whereas the yellow ranges are essentially random. Note that this happens both for narrow and broad response ranges. In view of the numbers it may not mean much.

Since the parameter α depends monotonically on V/d , whereas (in this experiment) V is fixed, we see that the participants apparently ignore the size d of the pictorial cube (varied over trials). It is as if they substitute a default angular size for the actual one (Figure 9).

However, Figure 8 somewhat misrepresents the data because there is a lot of interparticipant variation. This is explicit in Figure 9. Each participant uses a rather limited range of “default” values, a fact that is masked in the overall representations (Figure 8). There is quite a large range of default values. It is evident that many pairs of participants will likely disagree on what actually “looks like a cube!”

#### Epilog

The participants acted as if their distance was different from the actual distance. This can also be expressed in terms of angular extent, they tend to act as if the size of their angular field is different from what it actually is. From the individual median settings we can estimate this “default (pictorial) angular field.” The median value is 61∘ , the interquartile range is 48∘ – 78∘ .

The median value is compatible with the angular size of letter paper at normal reading distance. It is about the effect of a 30 mm focal length on a full-frame (Leica format, 24×36 mm) camera.

What is striking is the very large interparticipant range. Are people really that different? Apparently, yes.

Of course, as in all experimental phenomenology, there is no way to figure out what participants are really experiencing or what they are actually doing.

Some will experience no pictorial depth—“monocular stereopsis”—at all. Others may experience an inverted cube and be faced with a quandary. Some may use reflective thought and apply some (perhaps formally nonsensical) algorithms. Yet others may simply do something at random just to please us. All that will show up in the global measures.

Figure 9 is perhaps the most informative way to view the data. Here are some observations:

It seems unlikely that some people act fully at random, since there are no settings outside the veridical range.
Some people apparently apply a template, some fixed standard picture of a cube unrelated to the ratio of size to viewing distance: do they lack stereopsis? There is no way to find out.
Other people have a very wide range. Are they just sloppy? Here we have a test, the rank correlation between their settings and the actual values. Because we find not many rank correlations at the 5% level (see the orange bars in Figure 9) it seems likely that sloppiness often is a factor. However, there are notable exceptions.

The preferred perspective varies a lot among the participants. It is natural to wonder whether it is correlated with other measures, such as the extent of the visual field. We consider this issue later in the paper.

### Spatial Attitude in Pictorial Space

#### Concepts

As argued in the introduction—but focused on this task—it is important to distinguish between the *scene*, *visual space*, *picture*, and *pictorial space*.

Note that a physical scene is not necessarily implied, for instance when viewing paintings of heaven or hell. Visual objects are necessarily *actual*, but not necessarily *real*.^20^

The term “pictorial space” is often applied to the simultaneous structure of the pattern of pigments on the picture plane, then it is a 2D object. This is common with scientists who deny that stereopsis is possible without binocular disparity (Ames, 1925b; Claparède, 1904; Koenderink et al., 2011; Reid, 1769; Schlosberg, 1941).

It is also used for the imaginary space experienced when looking into a picture, then it is a 3D object. This is common with artists who close an eye when obtaining the *Fernbild* (iconic image) and think of binocular stereopsis as in bad (artistic) taste (Hildebrand, 1893).

In this report, we use “picture” for a physical object and “image” for imaginary, mental objects.^21^

It is common to reason about geometrical matters in both physical and mental spaces. This is permitted since formal geometry does not recognize the ontological distinction. However, one should be wary of “obvious” psychophysical relations. These are in no way “given” but are necessarily hypothetical.

#### Method

**Figure 10.**
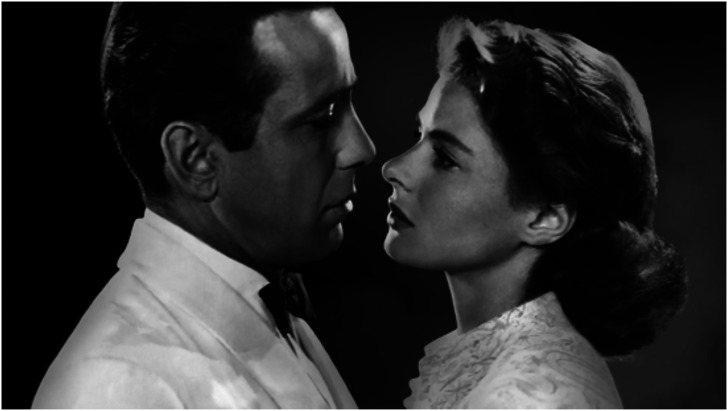
These are Humphrey Bogart and Ingrid Bergman in the movie “Casablanca,” 1942, directed by Michael Curtiz. Apparently, they look each other “straight in the eye.” (The picture shows a field of view of perhaps 20 ∘ –30 ∘ .)

The method is inspired by pictures such as Figure 10. Here the relative spatial attitude of the actors in pictorial space is very clearly defined.

**Figure 11.**
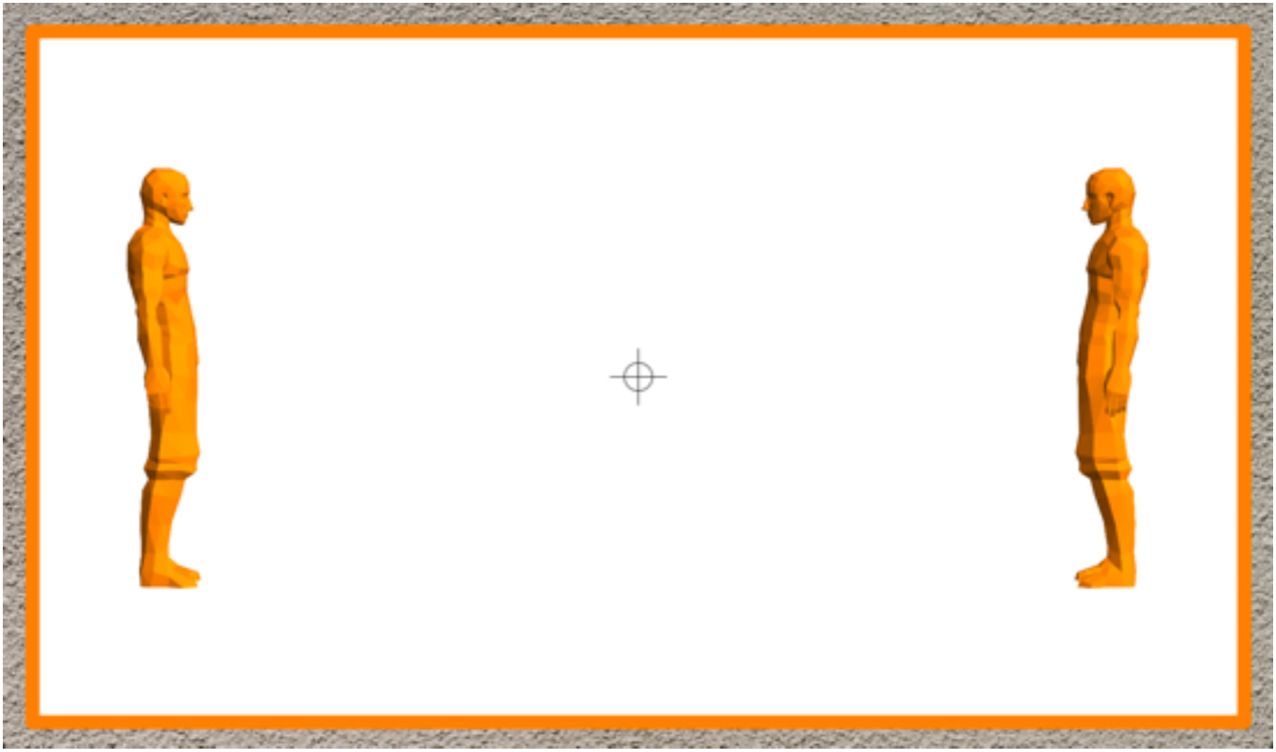
In the experiment, the participants rotate the actors in real-time (using computer graphics) so as to make it look like “they look each other straight in the eye.” Although a fixation mark was provided, participants were free to look back and forth between the puppets. (Note that this picture was viewed from a short distance, so the field of view was very wide.)

**Figure 12.**
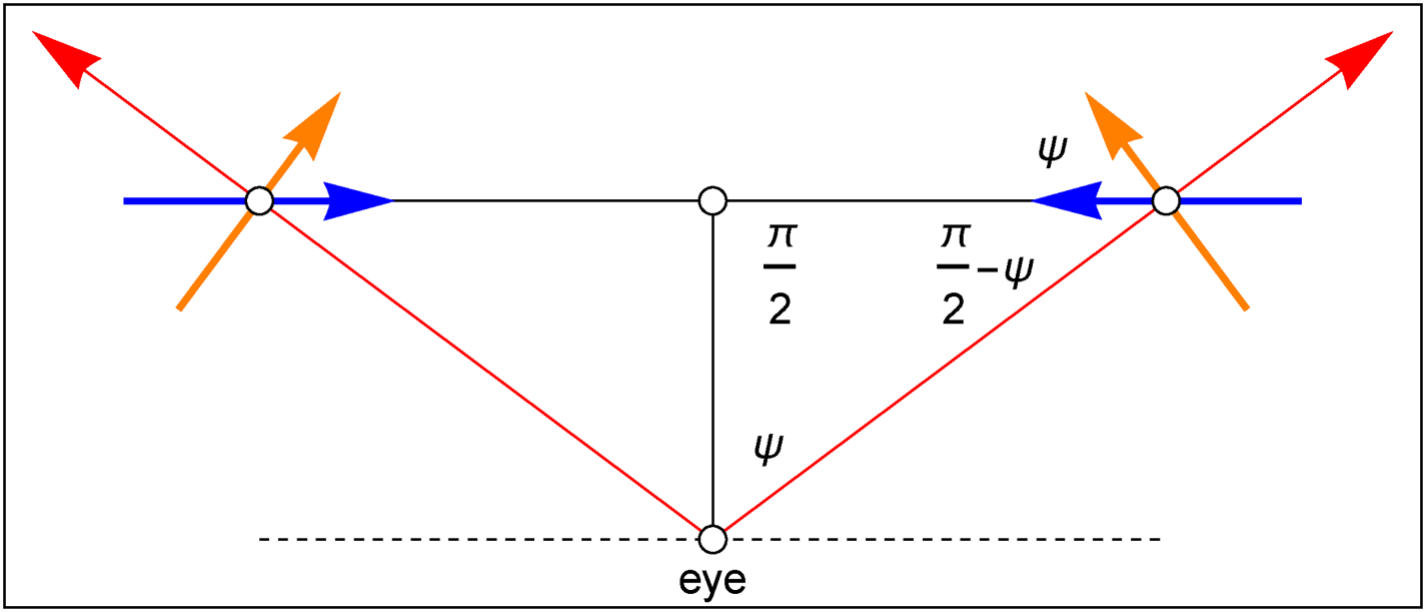
The relevant geometry of the experiment is simple trigonometry. The blue arrows show the spatial attitudes when the two puppets straightly face each other in a (hypothetical) physical scene. The orange arrows show the spatial attitudes when the two puppets are placed so as to obtain a picture as shown in Figure 10. The angular difference Ψ equals half the angular separation of the puppets in the (rather wide) fov. In this diagram, the “principal viewing direction” is vertically upwards, whereas the red arrows are “local visual rays.”

It is immediate to convert this into an experiment (Figure 11). Each participant performs a single trial once. The puppets were rigid and could be rotated about the vertical. In the “correct” attitude, the left puppet faces the right and the right puppet faces the left (thus “facing each other,” as in Figure 11). The single parameter ( ψ in Figure 12) rotates both puppets in synchrony, one clockwise, the other counterclockwise (Figure 12). The orientation of the puppets is readily seen in any attitude, as the body has a well-defined sagittal plane of bilateral symmetry. The session started with the puppet-pair in random orientation.

Viewing distance was 25 cm, yielding a FOV 121∘×90∘ .

##### Psychophysical correspondence

**Figure 13.**
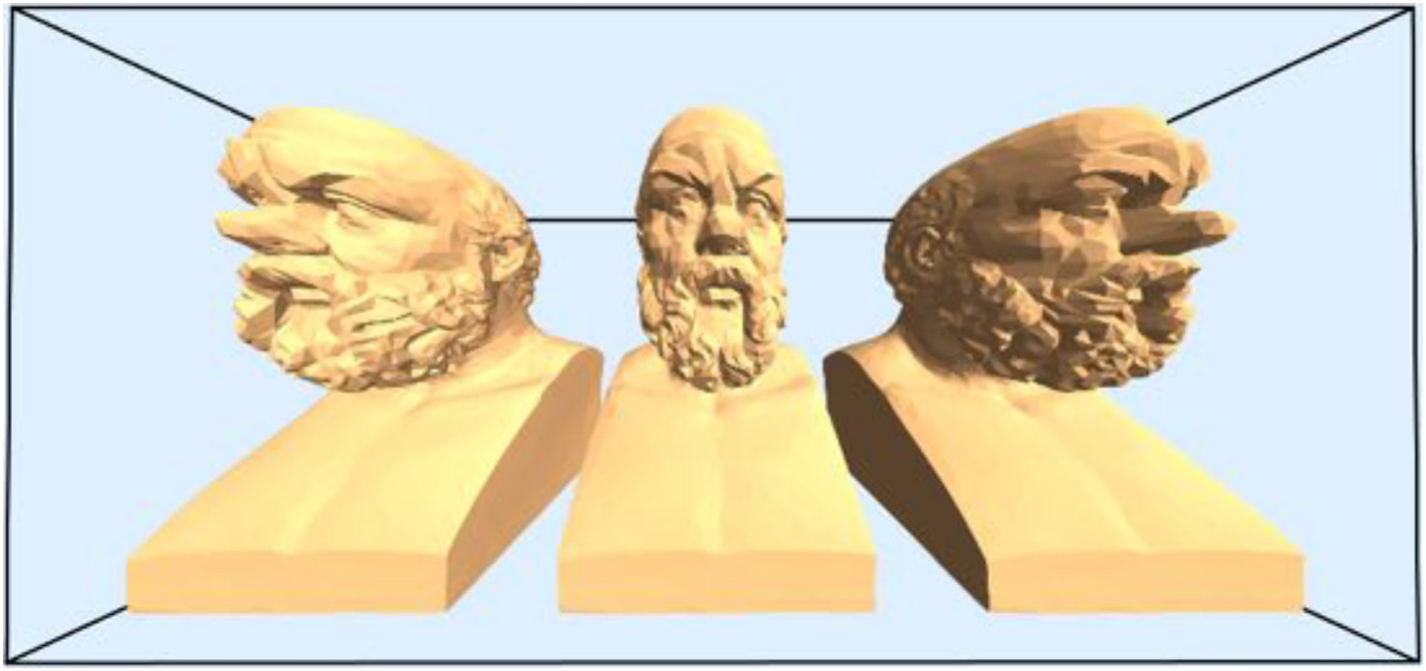
Three copies of a bust of Socrates placed perfectly parallel to each other and viewed frontally. The viewing distance is short, thus the field of view wide. (You probably view it from the “wrong” viewing distance. But it is not that you would not notice the effect from a “correct” viewing distance. In order to try you need to look at a greatly enlarged copy.) Note that the outermost busts “look rotated,” although they are not. For this pictorial effect to obtain the actual viewing distance of the picture has (perhaps surprisingly) hardly any influence. Artists intentionally “correct” for this type of pesky deformation due to linear perspective Pirenne (1970).

Figure 13 is a perfect perspective rendering of three identical objects in identical spatial attitudes. Many observers find it hard to convince themselves that the spatial attitudes are indeed identical.

A formal treatment of the experiment involves a psychophysical representation as shown in Figure 12.

#### Results

**Figure 14.**
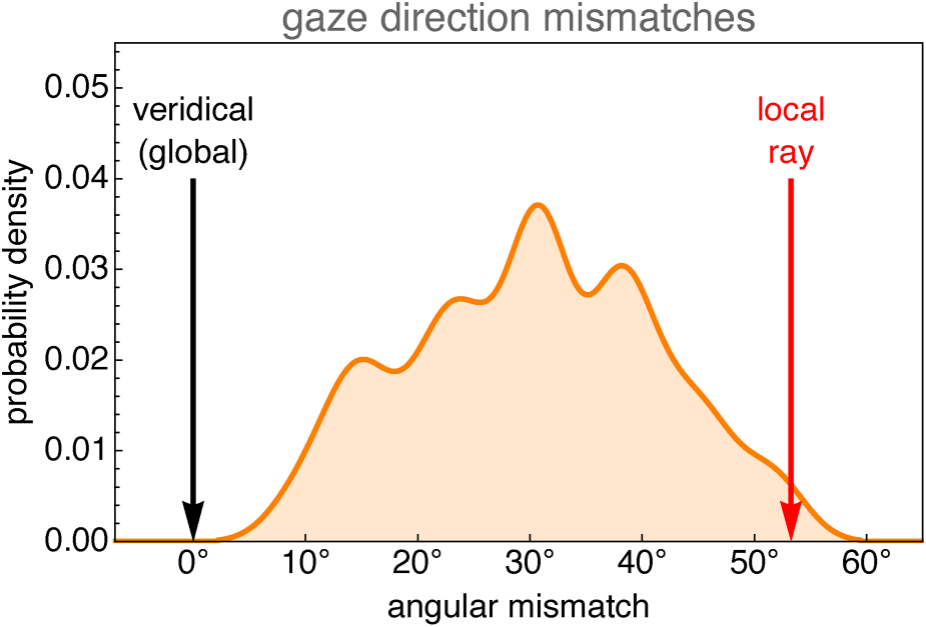
Smoothed distribution of mismatches (the equivalent of the angle Ψ in Figure 12).

We find that no participant sets a global, “veridical” configuration, whereas a few orient the puppets with respect to the local visual ray (see Figure 14). One might say that they act as if their visual rays were mutually parallel, which suggests that they treat the width of their visual field as 0∘ . Most participants are somewhere in between.

The null hypothesis that the data is distributed according to the uniform distribution on [8.28∘,53.32∘] is not rejected at the 5% level based on the Cramér–von Mises test.

On the whole, participants act as if the width of their vf was about half of that of the fov.

#### Epilog

In previous experiments of this type (Koenderink et al., 2010a), we found that most observers consistently orient objects with respect to the local visual ray. They routinely committed huge “errors,” up to more than 100∘ . In the present experiment, participants end up somewhere between that and a “veridical” setting.

The difference must be due to the precise nature of the task. In this experiment, we selected a rather singular spatial attitude, which—most likely—explains the difference.

### Pictorial Depth

#### Concepts

Despite the fact that there seems to be a consensus in vision science that “stereopsis” is a causal result of binocular disparity, many artists and viewers are convinced they look “into” pictures and experience “pictorial depth.”

It has been called “paradoxical monocular stereopsis” (Claparède, 1904; Ames, 1925b; Schlosberg, 1941), but is really the original meaning of “stereopsis” and considered a daily life experience in the arts.

The phenomenology is discussed in detail in Hildebrand’s (1893) book (see also Koenderink & van Doorn (2024)). The *Fernbild* is an iconic image that has “depth” as a quality that is experienced as spatial, although different from the picture-plane dimensions.

Several methods allow one to determine depth patterns (Koenderink et al., 2011). Although a mode of experimental phenomenology, this allows highly structured, quantitative responses to be obtained. It is not psychophysics, since it merely addresses imagery, not any physical entities.

In most cases, all pictorial objects lie behind the frontal plane (hence the name), but occasionally they “stick out” and break through the frontal plane. Considered doubtful taste in the visual arts, this is frequently done to add some spice to a picture. It works when used sparingly. Artists like to do it and viewers like to detect it.

#### Method

**Figure 15.**
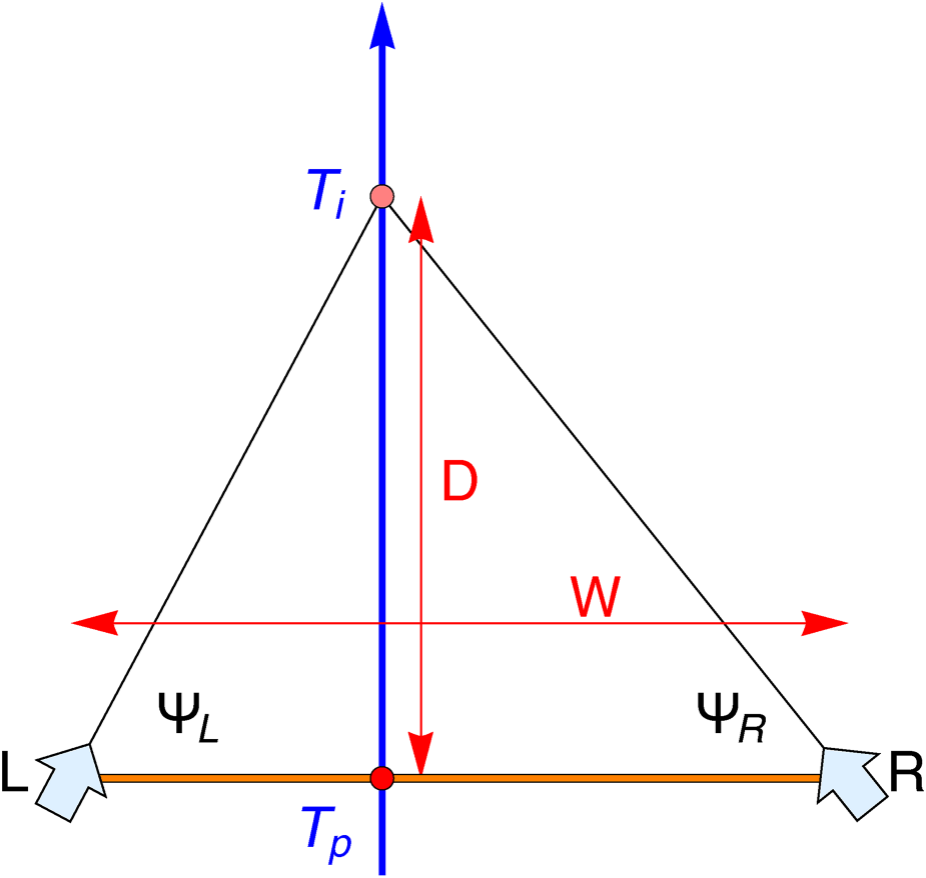
Geometry for the method of pointing. The participant controls the ratio D/W (W the picture width, D the “depth” of the imaginary target $T_{i}$ , the image of the physical point $T_{P}$ in the picture plane), the attitude $\Psi_{L}$ , $\Psi_{R}$ of the pointers L, R are adjusted in synchrony. Since W is “real,” whereas D is “imaginary,” their ratio is an oddity. It merely serves to drive the pointers, which are realistically represented on the screen.

We implemented a simple, fast method to indicate the depth of some target area in a picture. Although simple enough, the nature of the method is conceptually quite complicated (Figure 15).

**Figure 16.**
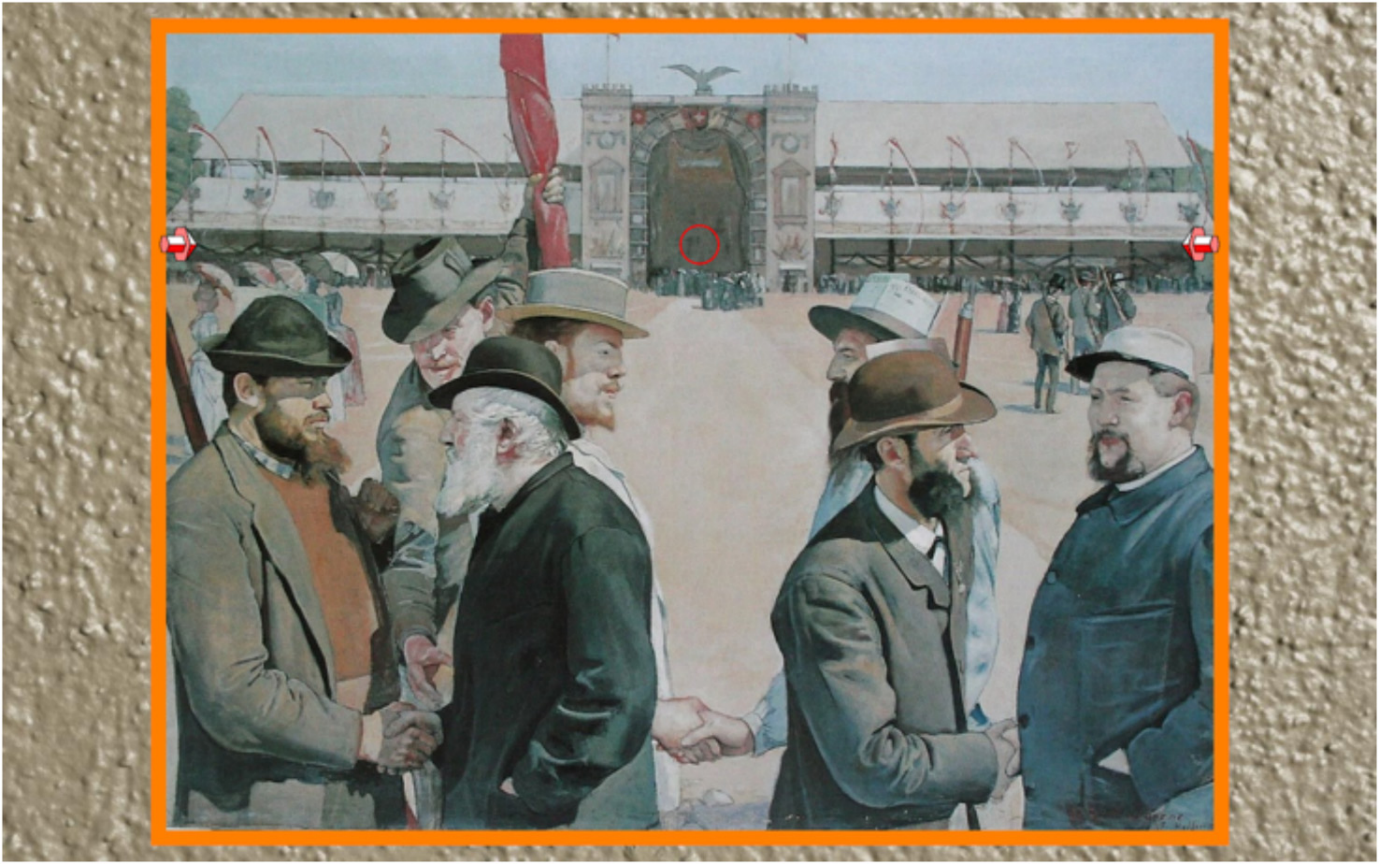
A screenshot of one of the stimuli. Note the arrows at left and right and the small red circle indicating the target area. The participant controls the inclination (see Figure 15) of the arrows. The pointers are always on the same horizontal level as the target. They slightly overlap the frame. Participants are meant to see them as in the picture surface, or frontal plane (as was mentioned to them). No one complained.

The picture plane is clearly indicated through the frame, which is superimposed on a wall surface, both displayed on the monitor. A target area is indicated by the smallish red circle. Although the circle is drawn on the picture plane, the indicated area is supposed to be in pictorial space. The two pointers are designed to look solid and unrelated to the picture. That is also why they slightly overlap the frame. One sees them as almost in the frontal plane, but really “outside of it.”^22^ The task is to have the 3D arrows point at the target area in picture space (see Figure 16).

**Figure 17.**
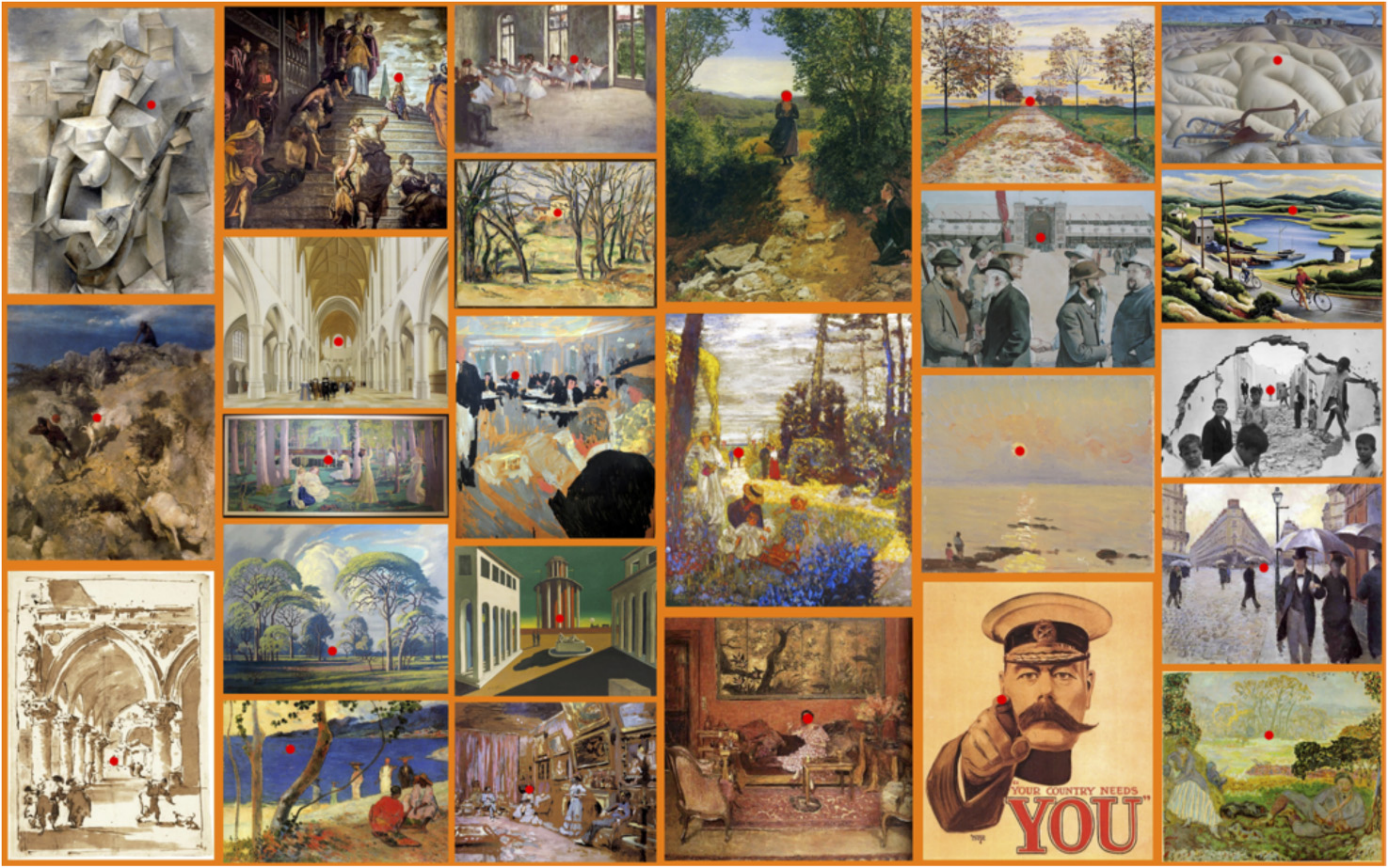
The pictures used in the pictorial depth task. The target locations are indicated by the red dots.

We used pictures with various amounts of pictorial depth (as informally judged by the authors), see Figure 17. We added one picture^23^ in which the artist intentionally (and big-time!) broke through the picture plane.

The viewing distance was 85 cm, yielding a FOV 55∘×33∘ .

#### Results

**Figure 18.**
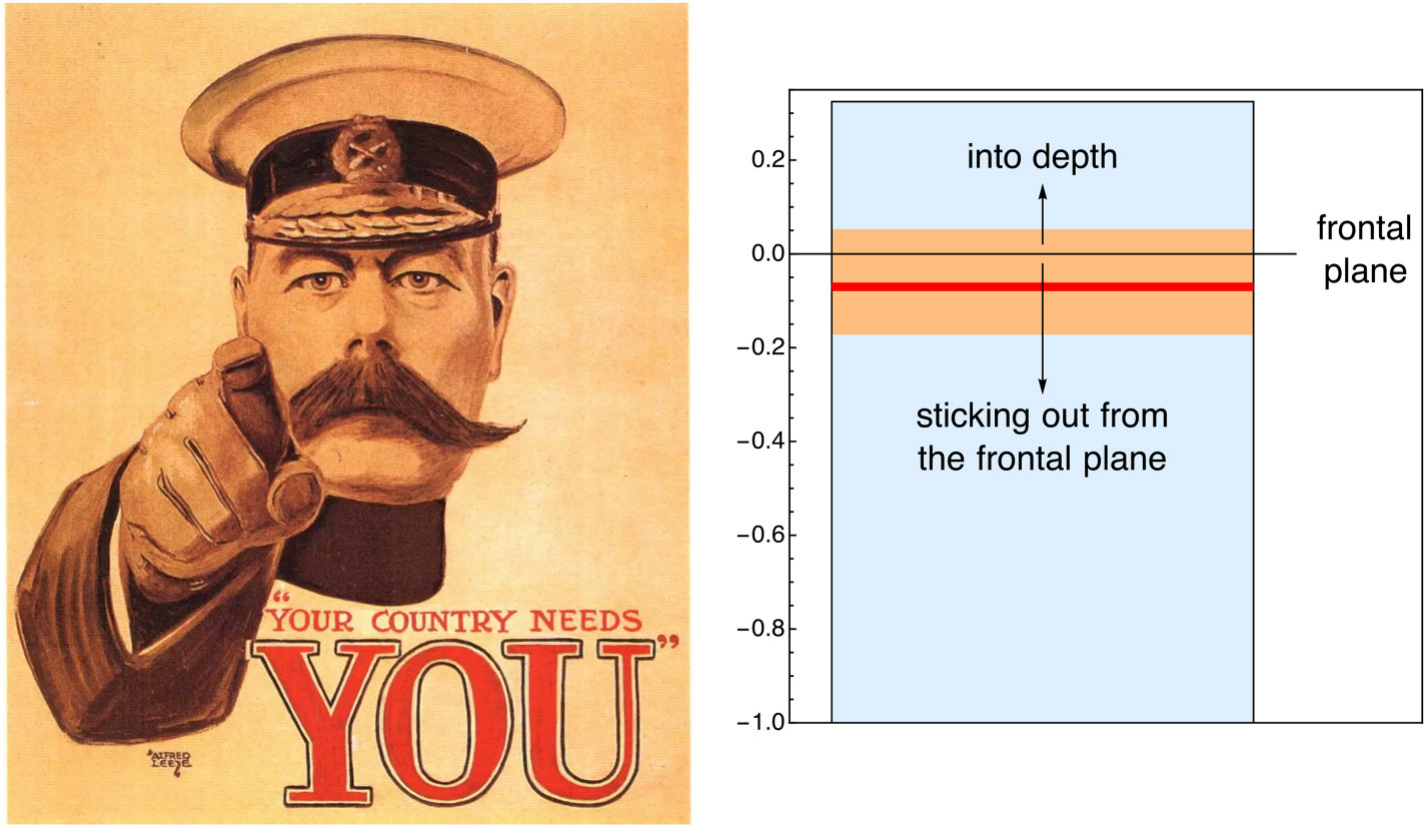
The Lord Kitchener example (left). At the right, we show the range, interquartile range, and median of the depth of the finger. Most participants see it break the frontal plane, but some don’t. Are the latter sloppy or do they “see” differently? No way to ascertain.

In most cases (a singular exception discussed below) the participants indicated depths behind the frontal plane. The Lord Kitchener example is—as expected—an exception (see Figure 18). Two-thirds (66%) of the participants indicated that Lord Kitchener’s finger broke the picture plane. The remaining third indicated a depth behind the picture plane, although of smaller amplitude.

The remaining analysis was done on the set of pictures with the Lord Kitchener picture left out.

**Figure 19.**
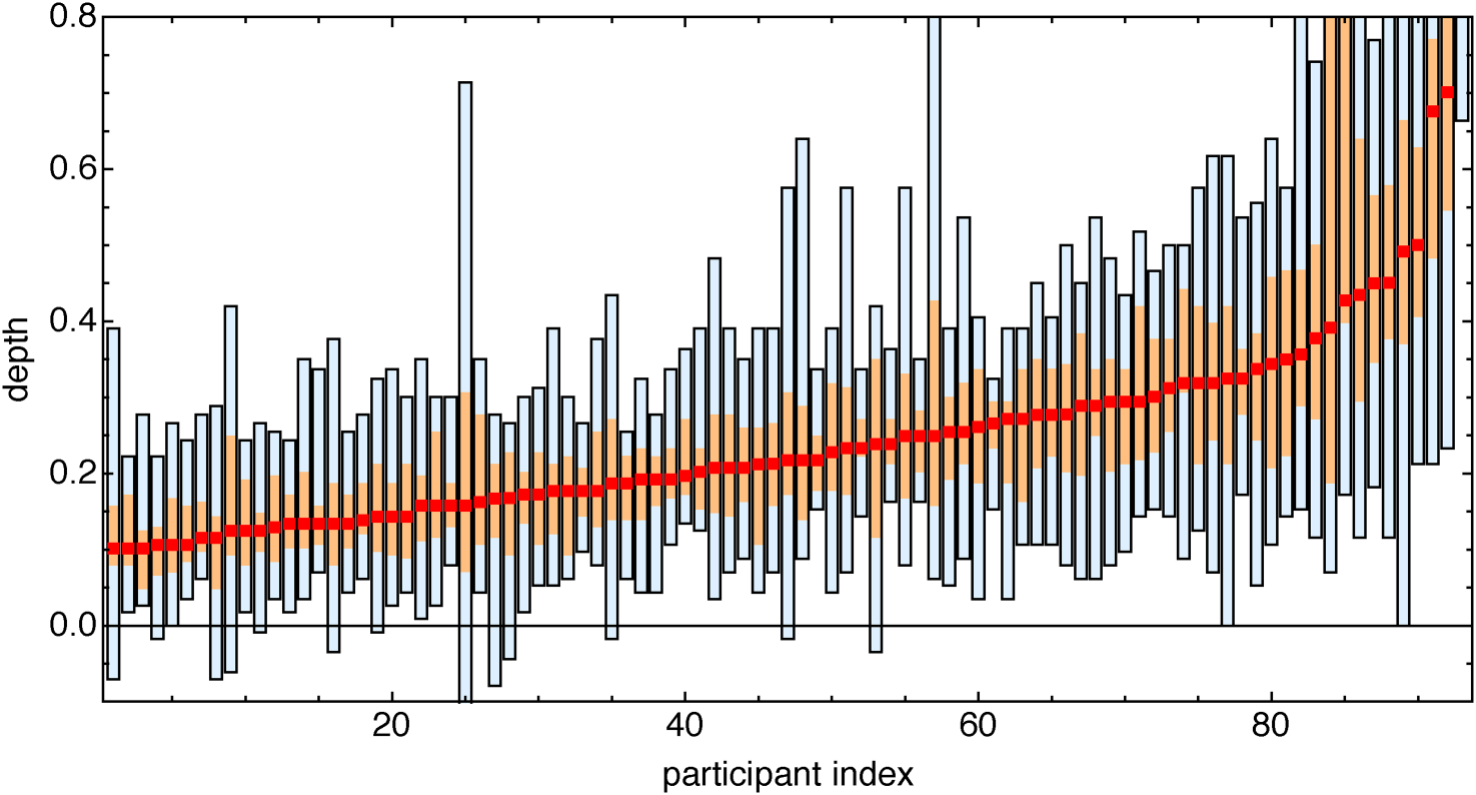
Range, interquartile range, and median of depth over all pictures except the Lord Kitchener picture, for all participants. (Compare Figure 20.) Note the huge variation over the group. One guesses that an extremely large range might indicate sloppiness, whereas extremely small interquartile ranges may suggest a lack of generic monocular stereopsis.

**Figure 20.**
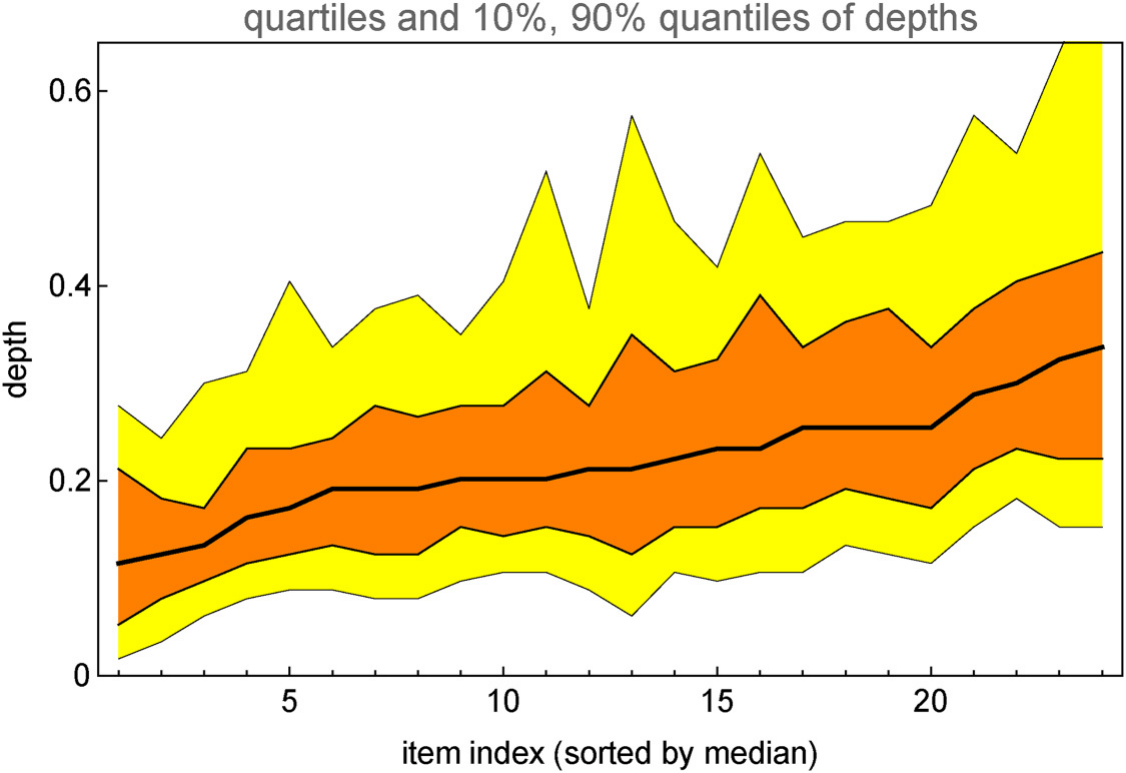
Distribution of depth over all items except the Lord Kitchener picture. (Compare Figure 19.) The black line is the median. The range is not indicated, it is much wider than the interquartile range.

We find rather large variations among the participants (see Figure 19). Note that this distribution is over all pictures. The distribution over all participants is shown in Figure 20. Thus the ranges and interquartile intervals do not (only) reflect the participant’s individual spread. There are some major idiosyncrasies.

One way to approach this problem is to compare all participants with the overall median. This is one reason why we picked pictures with rather different pictorial depths. This creates a pattern over stimuli that may be used as a benchmark to compare participants.

Kendall τ rank correlation with the median range from 0.12 to 0.71 (not all significant at the 5% level). As many as 24% of the participants fail to have a significant (at the 5% level) Kendall τ rank correlation with the overall pattern.

**Figure 21.**
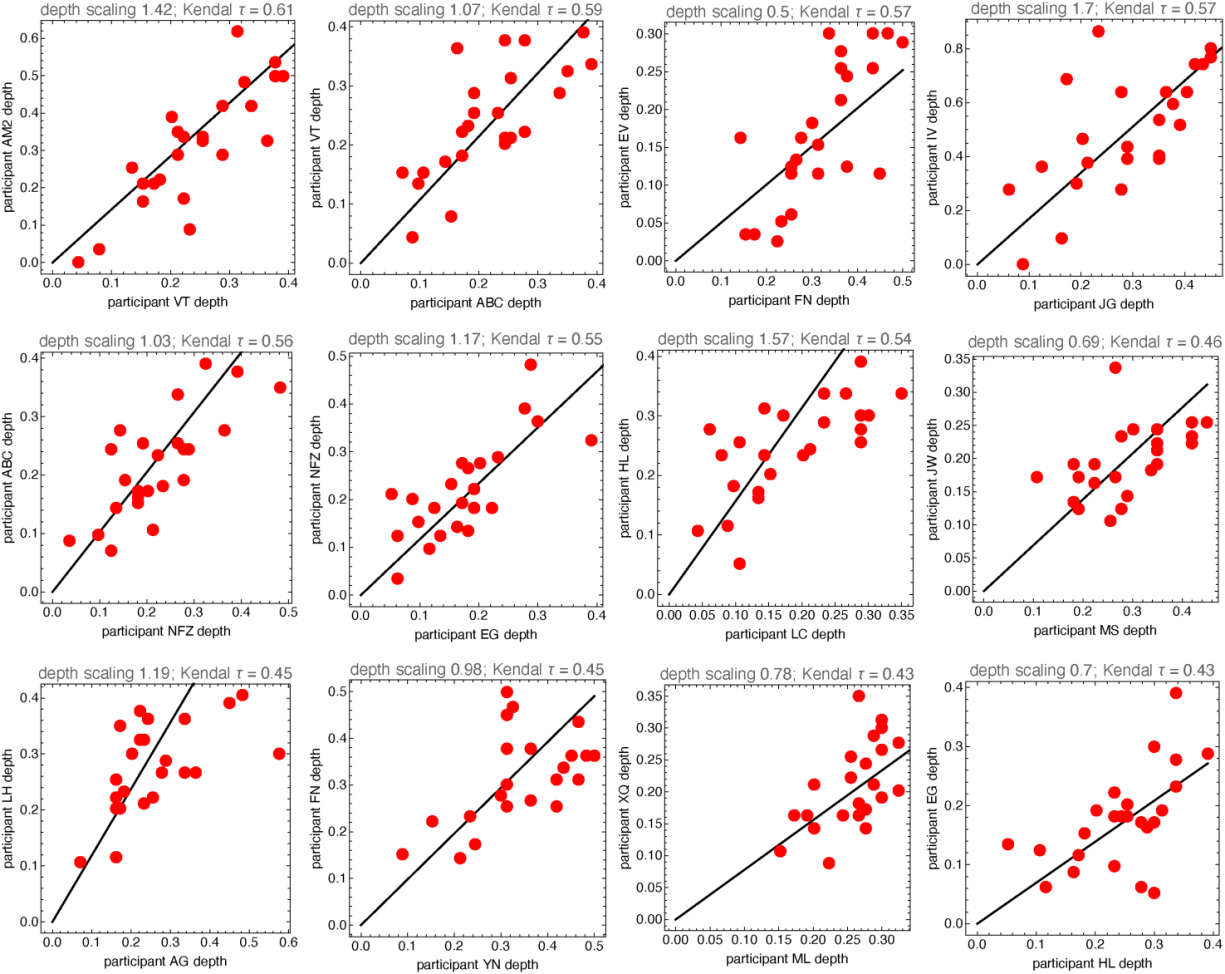
These are some list plots of the depths of pairs of participants that each correlate significantly with the overall median. Rank correlations are about 0.5 , so there is a lot of variation. That might be due to intraindividual factors (“sloppiness”) or interindividual differences. Since it is unlikely that all participants are sloppy the latter hypothesis is a viable option.

As one selects participants that correlate well with the overall median, one finds that they also correlate well with each other. We show examples of such pair-wise comparisons in Figure 21.

**Figure 22.**
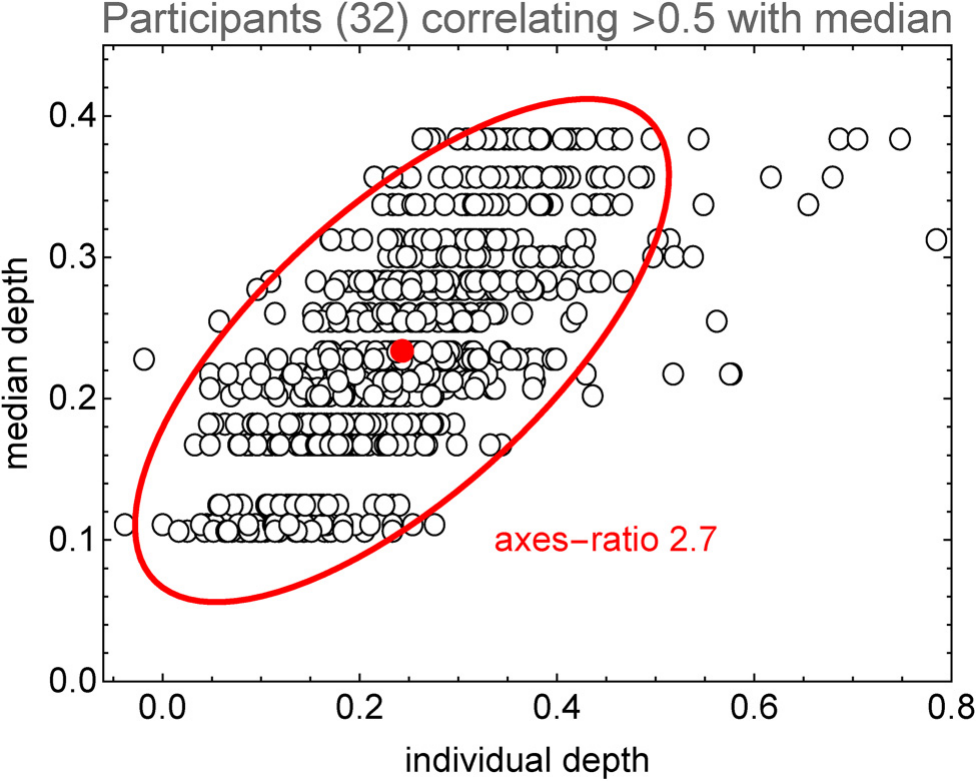
A scatterplot comparing all participants that individually (rank-) correlate better than 0.5 with the population median. We have scaled the individual data to let their median depth fit the median of the overall median depth profile. There are 32 participants in this group. The 95% covariance ellipse has an axis ratio of 2.7. Thus, the group as a whole does not do much better than discriminating foreground, middleground, background, and (perhaps) a backdrop, see Figure 23. (Of course, individual participants are likely to do much better.)

**Figure 23.**
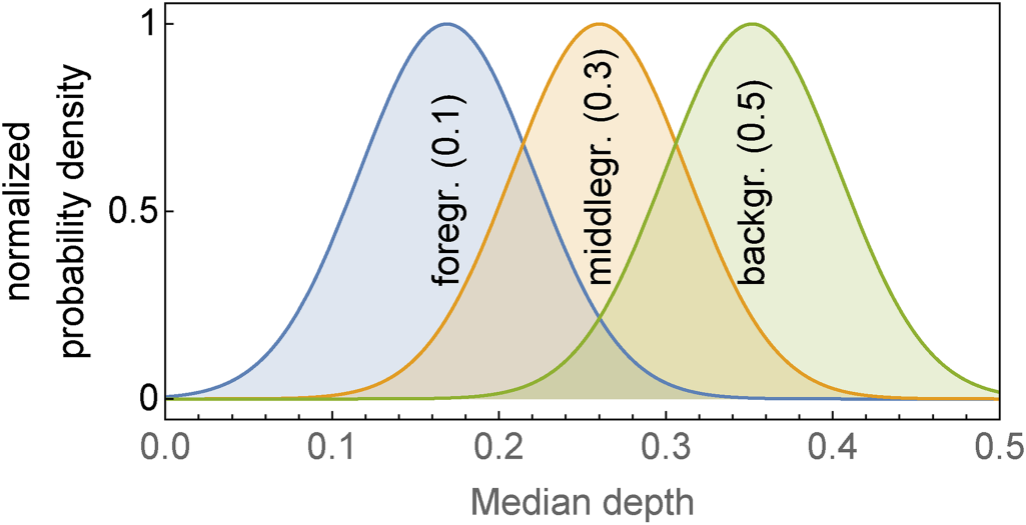
Three sections of the 2D probability density function shown as the covariance ellipse in Figure 22, normalized to the same height in order to allow easy comparison. The sections are at “individual depth” 0.1 (“foreground”), 0.3 (“middleground”), and 0.5 (“background”). The distributions are seen to overlap fairly strongly on the common “median depth” scale. (The discriminability index ( $d^{\prime }$ ) for the differentiation between two adjacent levels is about 1.8 .) Thus the population barely discriminates the three depth levels foreground, middleground, and background (see Figure 22).

One may attempt to somehow “normalize” the individual depth distributions and pool all (or many) participants (Figure 22). We select the participants with rank order correlation over 0.5 with the median response (these are all significant). The resulting distribution is nonisotropic, and the axes ratio is 2.7. This nonisotropy is rather low, suggesting that this population as a whole “resolves” perhaps three depth levels (Figure 23).

Such a depth resolution is far less than what we routinely find with more sophisticated methods, where we find resolutions up to a hundred depth levels.

#### Epilog

**Figure 24.**
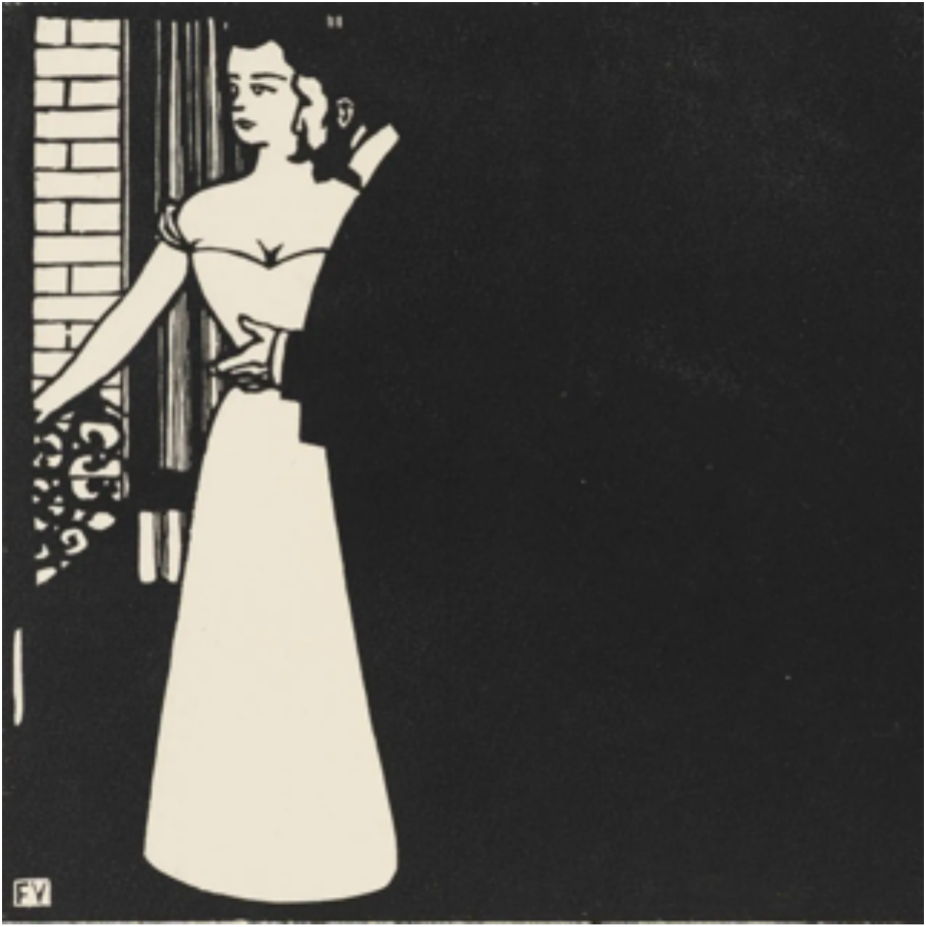
*“A man in front of a woman, both in front of a wall”*—is this a judgment in reflective thought, or an intuition (monocular stereopsis)? In this case, cognitive judgment will do. An iconic view yields a more complete spatial atmosphere, but is not needed for this kind of judgments. People depending on cognitive judgments would protest when you suggest they lack (monocular) stereopsis. Moreover, like many scientists, they would not understand what the issue is about. (Woodcut by Félix Vallotton.)

Although virtually anyone can “see” that a person stands in front of a house in a photographic snapshot, this may well be a cognitive judgment based on overlap (occlusion), perhaps even in reflective thought (Figure 24). However, pictorial depth proper evidently occurs at the sentient level of the creative imagination.

A differentiation between sentience and sapience is not possible in an objective (scientific) sense. We should reckon with the possibility that part of the population might lack monocular stereopsis in the relevant sense.

Consider the cases in which Lord Kitchener fails to break the frontal plane and the cases of insignificant rank correlation with the population median. Both are possible indications that the observer might be weak in monocular stereopsis. As it turns out to be the case, these cases especially often co-occur (Jaccard index 30, 5% level for the Jaccard index is 24). Moreover, this group of participants contains predominantly slow responders (Jaccard index 17, 5% level 16), which might indicate uncertainty with the task or processes involving reflective thought.

Such are about the only available criteria, for there is no such a thing as “veridical depth” to assess exceptions. Moreover, it is perfectly acceptable that people come in mutually very different varieties. Estimates from experimental phenomenology should be of some interest in understanding frictions in person-to-person interactions when discussing a painting.

A further investigation should involve results from different types of experiments. We consider that in a later section.

### Feeling of “Presence”

#### Concepts

Sometimes one has the feeling that one might “step into the picture.” This is most common when the FOV is very wide. However, it also depends on pictorial content.

This effect might well be related to the experience many people have with snapshots that capture the *impressive view* of mountains. They tend to be disappointed with the resulting pictorial objects. Very often the majestic mountain comes out like a mole hill. At least with naive snapshots, for artists know how to deal with the problem.

It is common to find that important objects are enlarged in their depiction (Baldwin et al., 2014; Burleigh et al., 2018; Pepperell & Haertel, 2014; Ruta et al., 2016). This leads to a more satisfactory view. The feeling of “presence is heightened.”

#### Method

**Figure 25.**
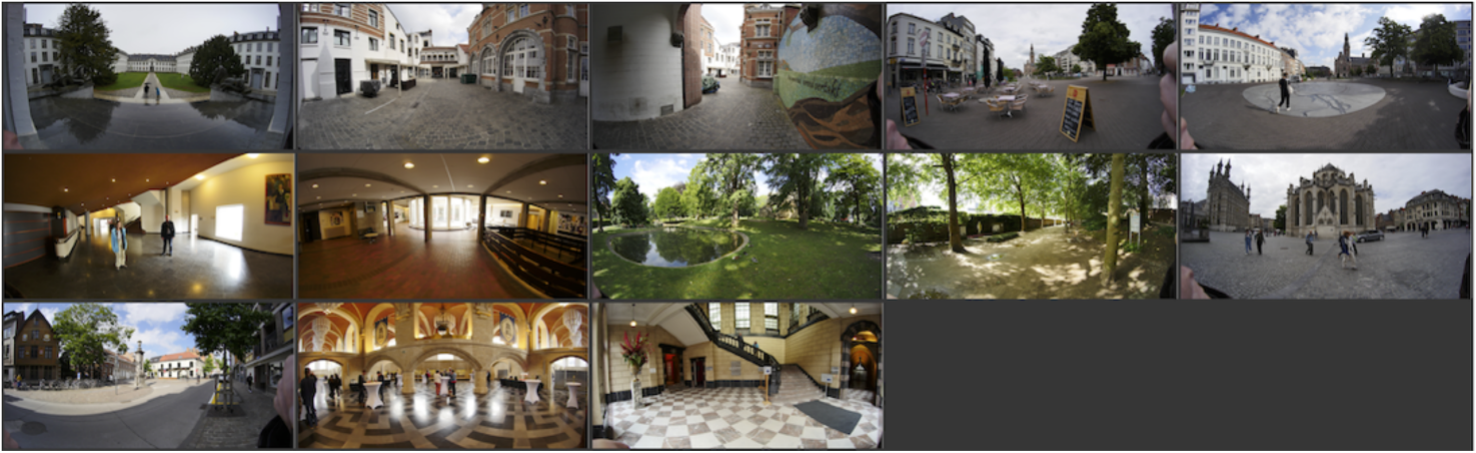
Pictures used in the “feeling of presence” task.

We presented 180∘ panoramas of scenes as equirectangular maps (Figure 25). The scenes were familiar to most participants (some well-known scenes within the city center of Leuven, as well as indoor scenes of University buildings that are open to the public). The participants had real-time control of a local scaling of the central region. Of course, that implies an opposite scaling of the marginal regions since the depicted scene always contained the same material.

**Figure 26.**
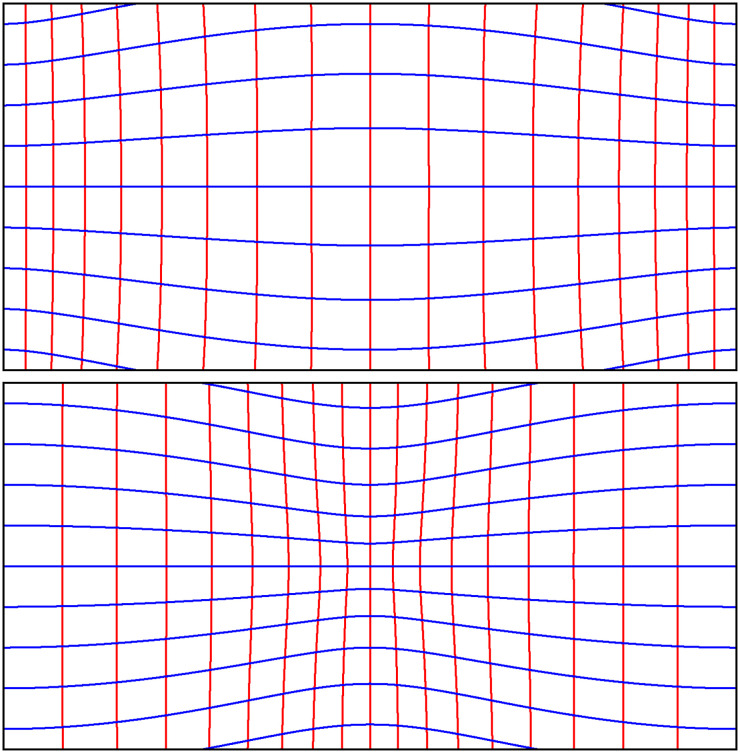
Examples of the nonlinear warping in the “presence” task. At the top is a central dilation, at the bottom a central contraction. The red curves are loci of constant azimuth (“verticals”), while the blue curves are constant elevation in the visual field. (Note that loci of constant elevation are “‘horizontals” in some definitions, but not in others! Straight lines in space map on great circles in the visual field, which map on straight lines in linear perspective, but not necessarily in the equirectangular map used here.) The panoramic extent (horizontal) is 180∘ , and the elevation (vertical) ranges between ±45∘ . Such pictures tend to look “natural” to the authors. In the case of pictures of the natural environment, nobody complains. In contradistinction, if there are important “horizontals” in scenes complaints grow beyond reasonable bounds. Thus, scenes containing architectural detail are problematic. Guido Hauck’s observations on the “collinear infection” are very relevant today. In the (not warped) equirectangular map, verticals are depicted straight and vertical, but horizontals appear “curved.” Several of our participants loudly complained. It severely affected their settings. Perhaps a reason to distrust the results from this task.

We used a nonlinear magnification factor (Figure 26). (Note that the warped representations are no longer “equirectangular!”) The parameter was controlled by the participant. The task was to set the values such as to create a rendering that elicited the maximum feeling of “presence,” or “being immersed in the scene.”

The viewing distance was 85 cm, yielding a FOV 55∘×33∘ .

#### Results

**Figure 27.**
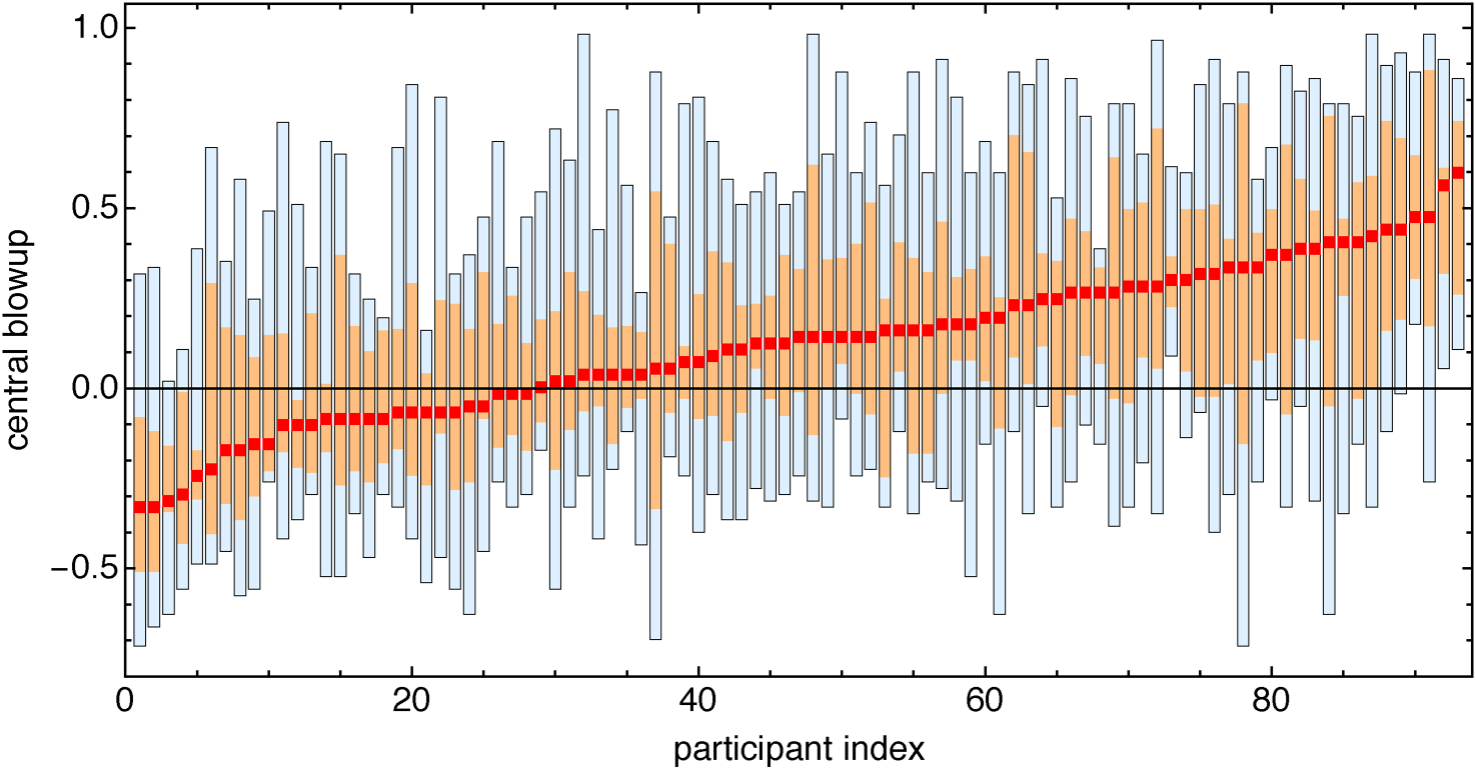
Distribution of participant settings over all stimuli. The parameter is positive for central enlargements, and negative for minifications. (Compare Figure 28.)

**Figure 28.**
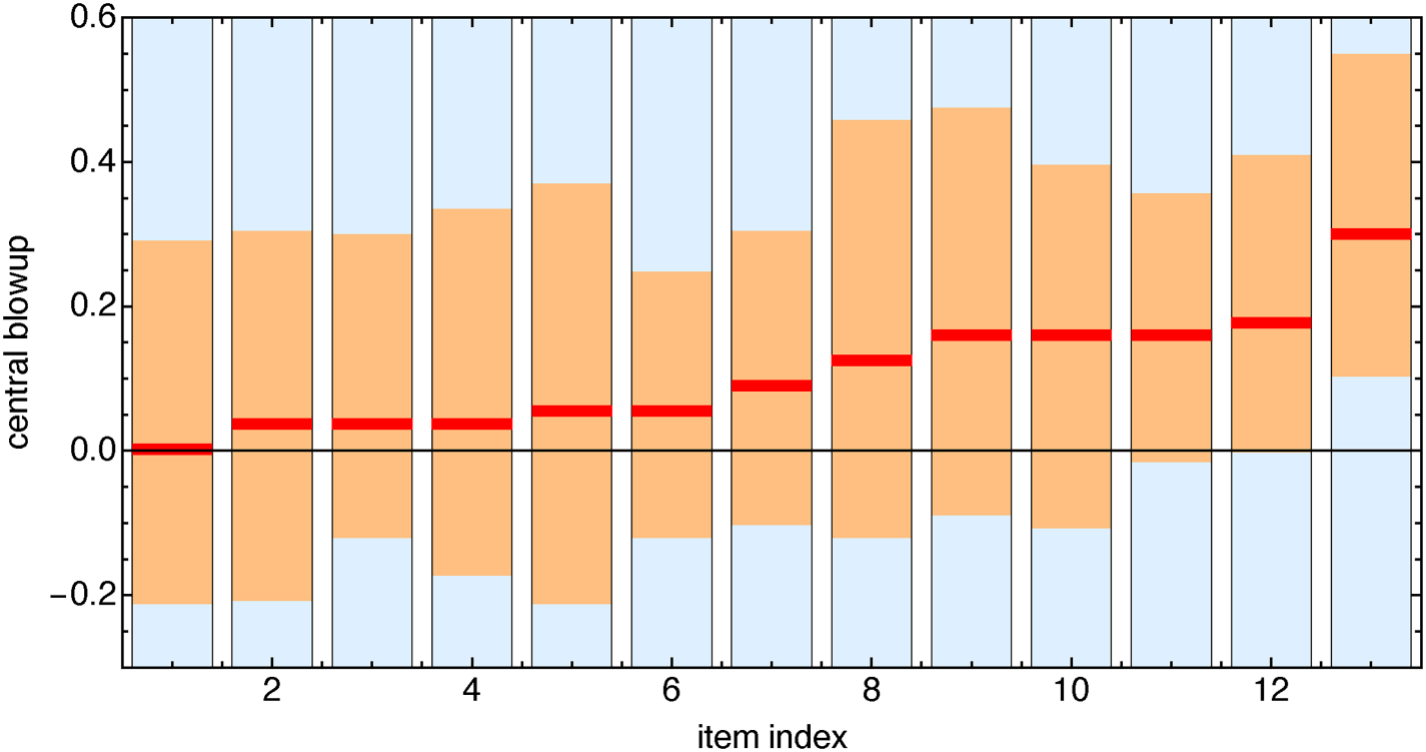
Distribution for stimuli over all participants. (Compare Figure 27.)

Two-thirds (68%) of the settings reveal a magnification of the central area (see Figures 27 and 28).

**Figure 29.**
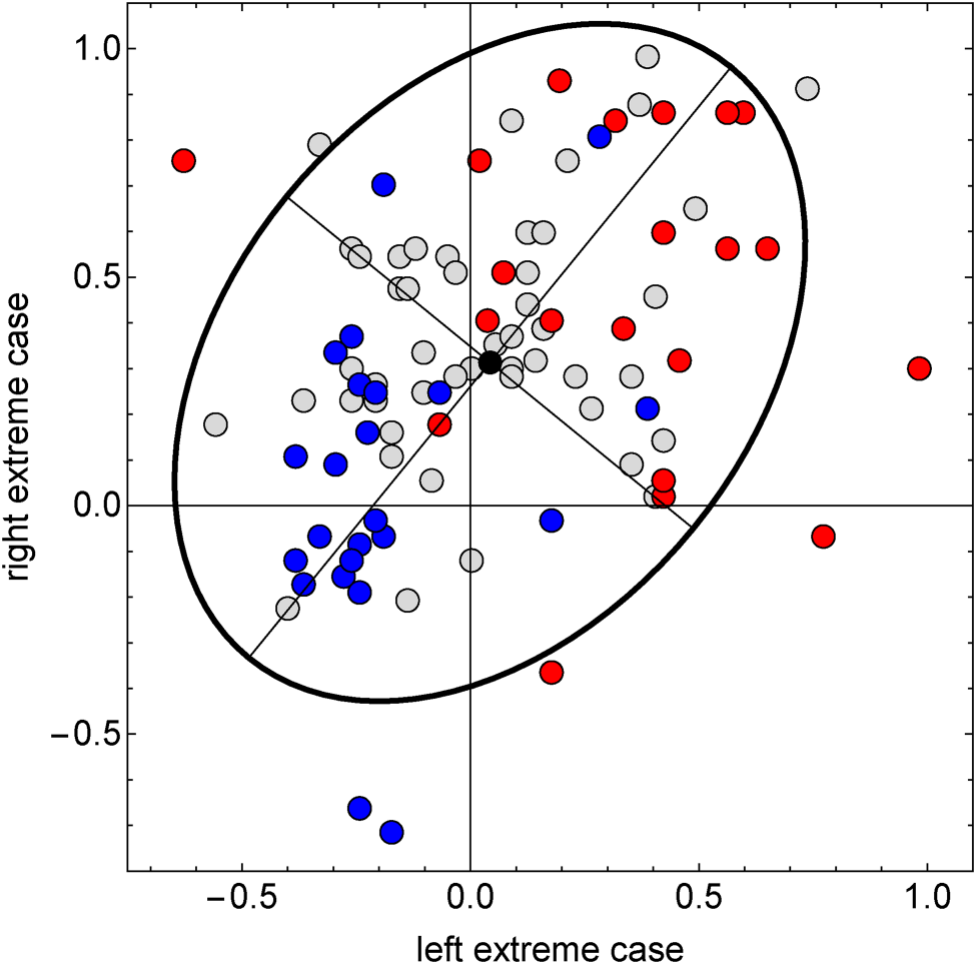
Scatterplot of settings for all observers for the two extreme stimuli (horizontally Figure 30 bottom, vertically Figure 30 top). The 25% observers that scored consistently high over all stimuli are colored red, the 25% that scored consistently low over all stimuli are colored blue, and the others gray. The ellipse is the 90% covariance region. The null hypothesis that the correlation coefficient is equal to 0 is rejected at the 5% level ( p value = .0005). There is at least some structure, but the scatter is high.

This is the main result of this task. The scatter in the responses is very high and precludes in-depth analysis. We show results for the two stimuli that led (going by median setting) to the most extreme values (Figures 29 through 31).

**Figure 30.**
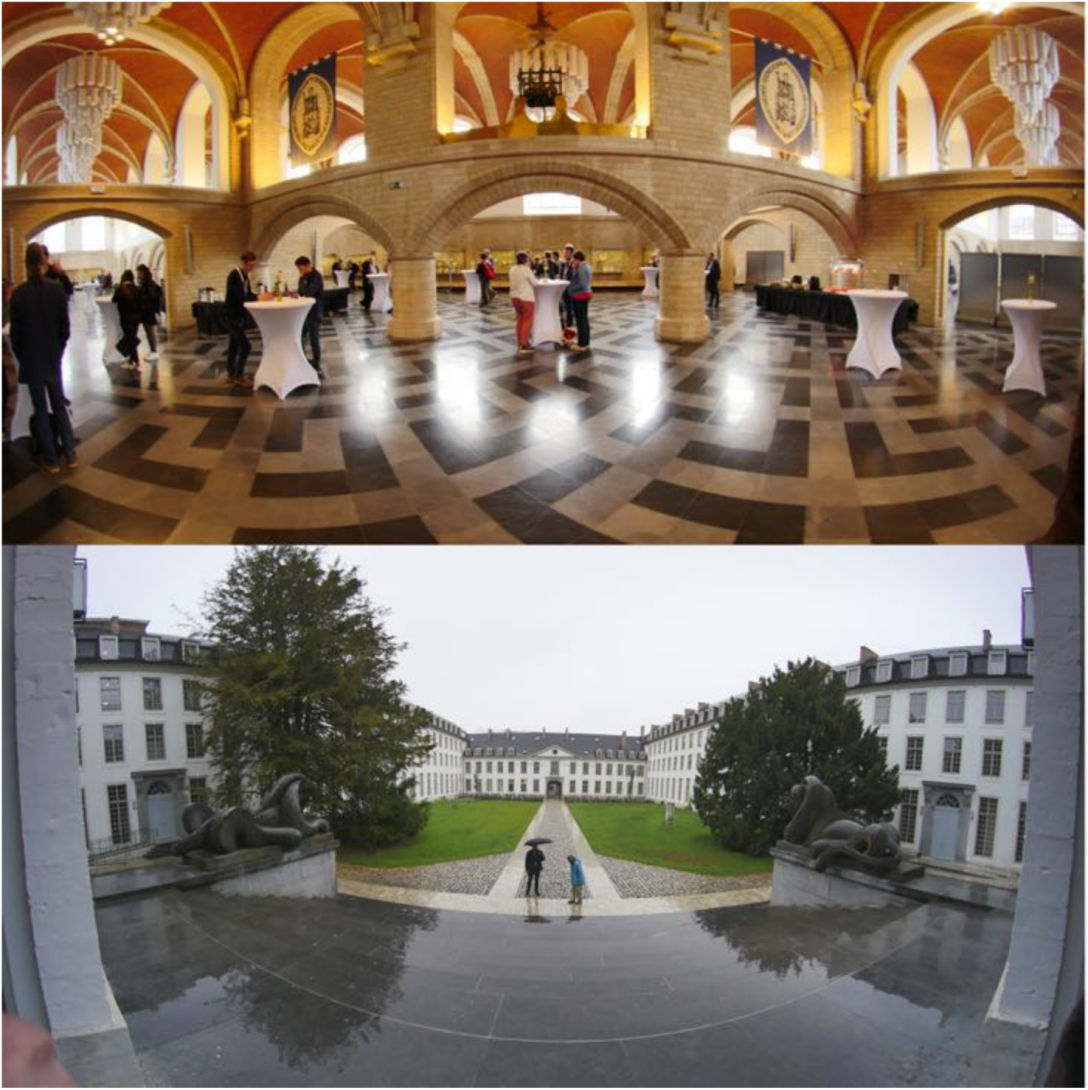
The originals for the two stimuli lead to extreme results.

**Figure 31.**
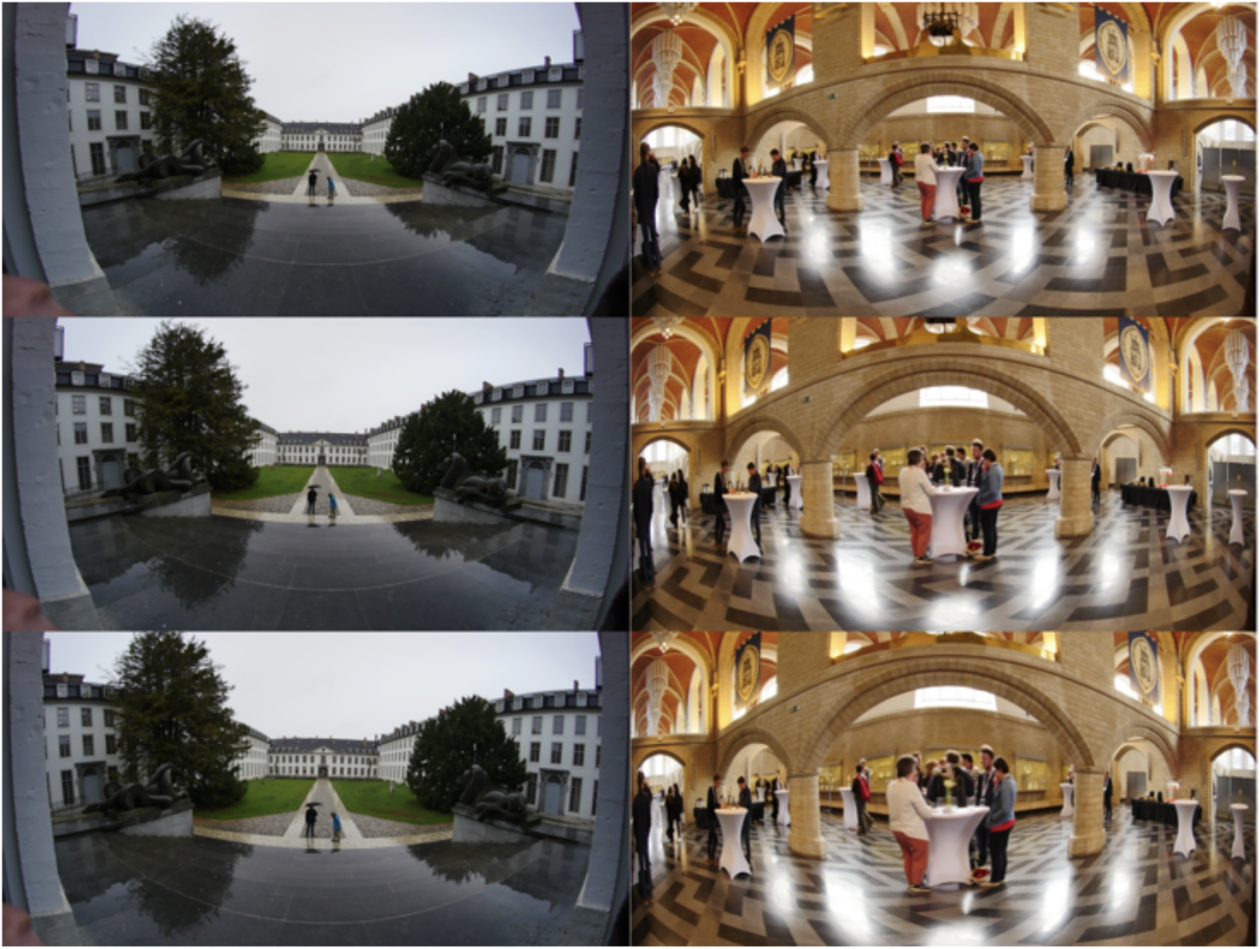
The two stimuli leading to extreme results. In each case, we show the quartile values (see Figure 27). The originals (see Figure 30) are outside the interquartile range in both cases.

There is no participant- pair that yields a significant correlation (at the 5% level). Only a few participants correlate with the overall median. Idiosyncratic interpretation of the task seems a likely cause. This is one cause of interindividual differences that we need to reckon with anyway. However, it is a cause that is not necessarily connected with spatial abilities.

#### Epilog

In postsession debriefings, we obtained a variety of remarks that made us doubt the value of the present experiment. Given its potential relevance, this is to be deplored. Reasons appear to be both the formulation of the task and the nature of the stimuli.

What is a good criterion? We tried “presence” (“do you feel you can step into the picture?”). We avoided “which looks more natural?” because scaling a local area necessarily introduces deformations, something many people can’t stand.

A good example of the latter is common complaints about the curvature of edges that “should be straight.” This problem was already noted by Guido Hauck, who spoke of a “collinear infection,” especially endemic among academics (Hauck, 1879). Indeed, the bulk of our participants appear to suffer from this syndrome. As Hauck already noted *Naturmenschen* (paradisiacal hominids) are rare today. However, it is something connected with spatial abilities, thus it might help indicate one dimension of differences in correlations over tasks.

A likely antidote might be the avoidance of obvious architectural details and to focus on the natural environment.^24^

A rephrasing of the formal task might possibly help too.

### Aspect Ratio

#### Concepts

One important property of a picture is its aspect ratio. “Aspect ratio” refers to the ratio of the width to the height of a rectangular picture plane, or frame.

Common aspect ratios range from “cinematic” (about 12:5 ) to 1:1 (e.g., 6×6 cm Rollei negative), with “normal” values from 3:2 ( 36×24 mm Leica format on 35 mm film) to 4:3 (tv, microfourthirds chips). In continental Europe, the $\sqrt{2}$ format (about 7:5) is perhaps most often encountered as the aspect ratio of common letter paper.^25^

We prefer to use the dual logarithm of the width to height ratio. The advantage is that portrait and landscape aspects get the same absolute number. The square 1:1 maps on zero. A sheet of European letter paper gets +0.5 in “landscape aspect,” −0.5 in “portrait aspect.” One soon gets used to this convenient measure.

In the arts far more extreme aspect ratios are encountered, although sparsely. Both artists and viewers have expectations and preferences.

This is a topic that has nothing to do with 3D, but only with 2D pictorial space. It is a topic that cannot be omitted in a broad investigation of pictorial properties and preferences.

#### Method

Participants had real-time control over the displayed aspect ratio of pictures. They were instructed to adjust to their preference (for the given picture) but abstract from possible prior acquaintance (this turned out to be rare) with the pictures.

Expected tendencies are a regression toward a preferred standard and a wish to let pictorial objects look “normal.”

**Figure 32.**
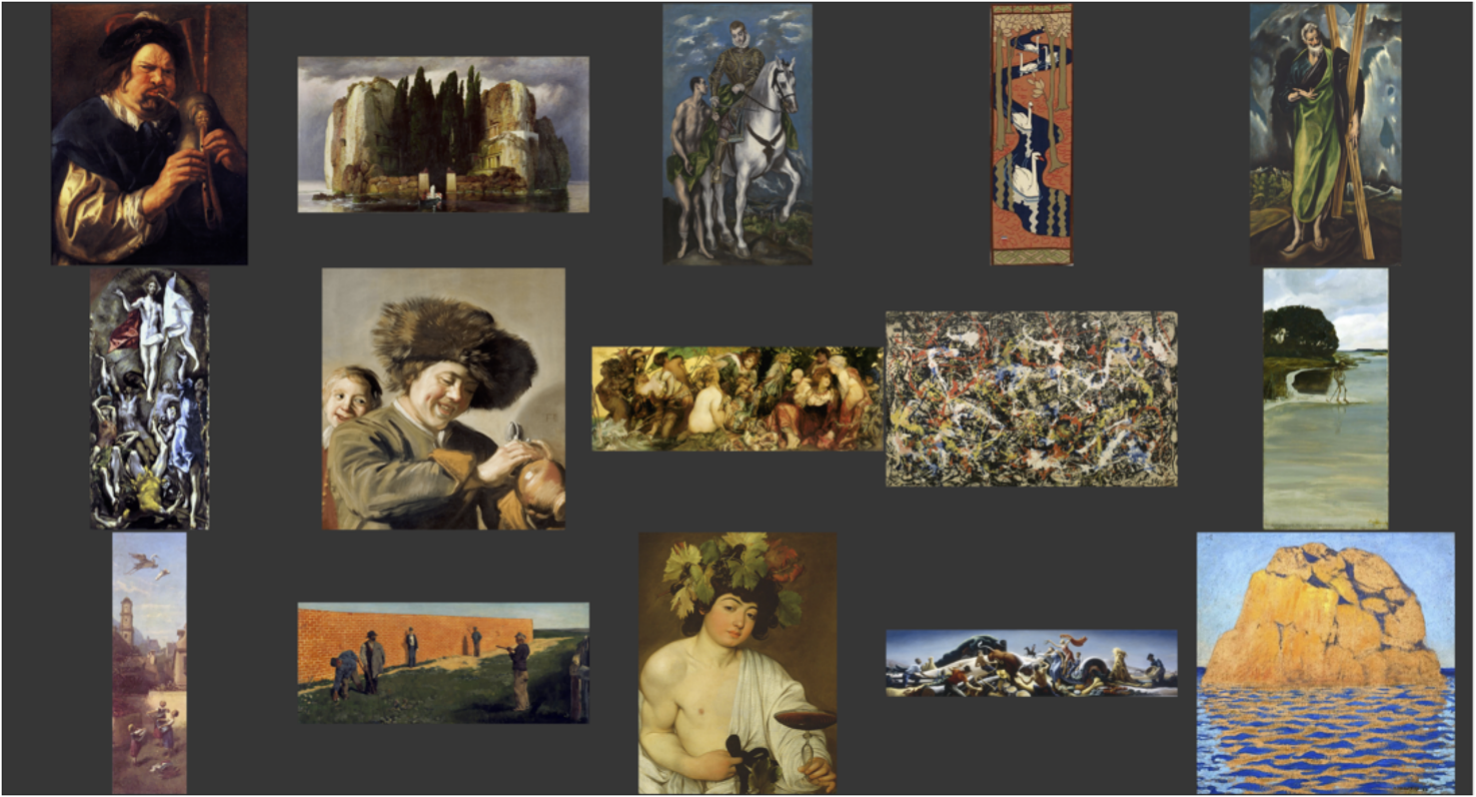
Pictures used in the aspectratio task.

So we collected a wide range of aspect ratios and topics that contained various cues to “normality.” We then added three pictures by El Greco, because it seemed a priori likely that participants might want to “correct” them (Firestone, 2013). (See Figure 32.)

The viewing distance was 85 cm, yielding a FOV 55∘×33∘ .

#### Results

**Figure 33.**
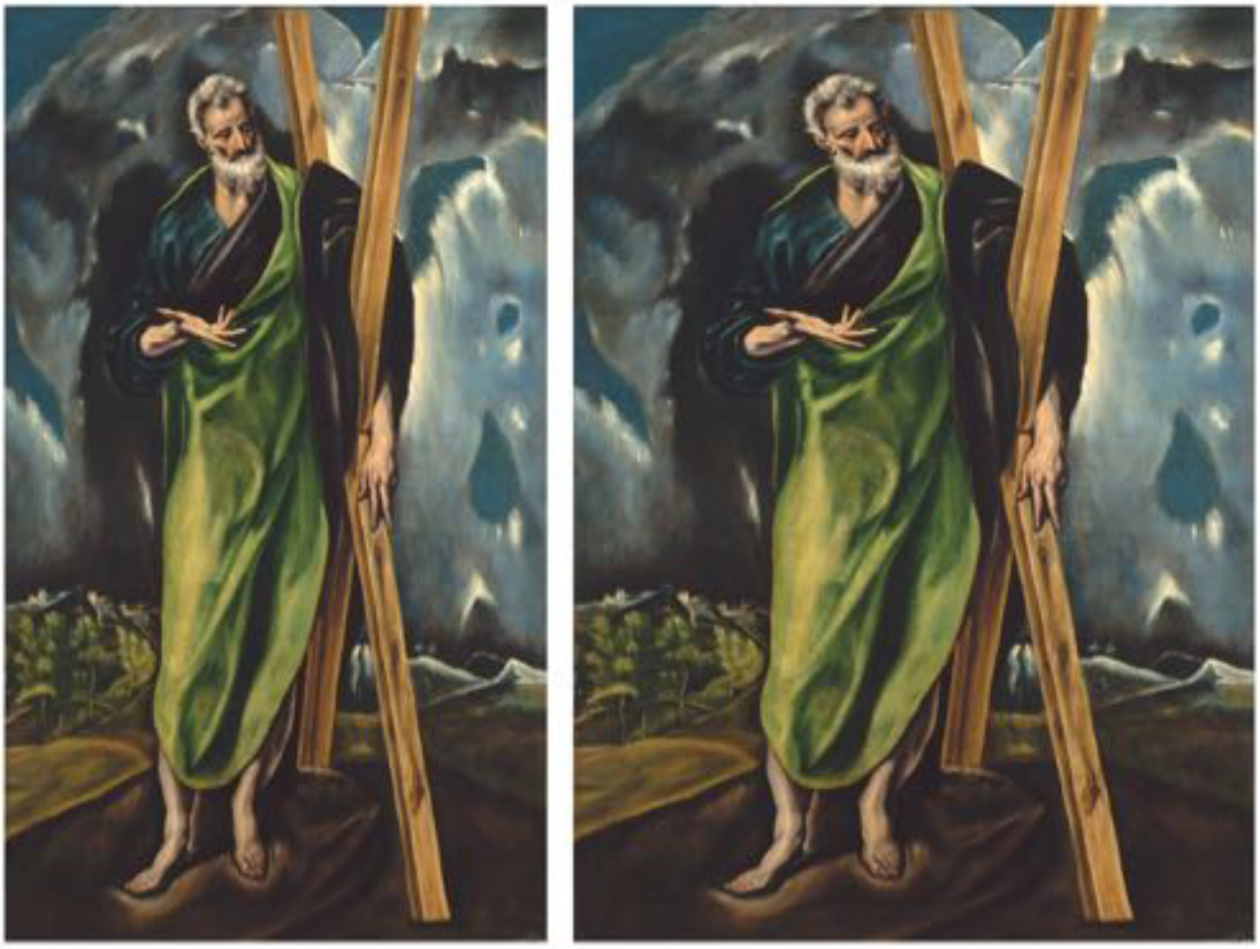
Typical “correction” of an El Greco painting. Here we used the median over all participants. Original at left.

As expected, 94.4% of the El Greco settings were “corrected.” There was no significant correlation between any pair of the three El Greco paintings. The corrections were appreciable (see Figure 33). In the further analysis, we omitted the El Greco samples.

**Figure 34.**
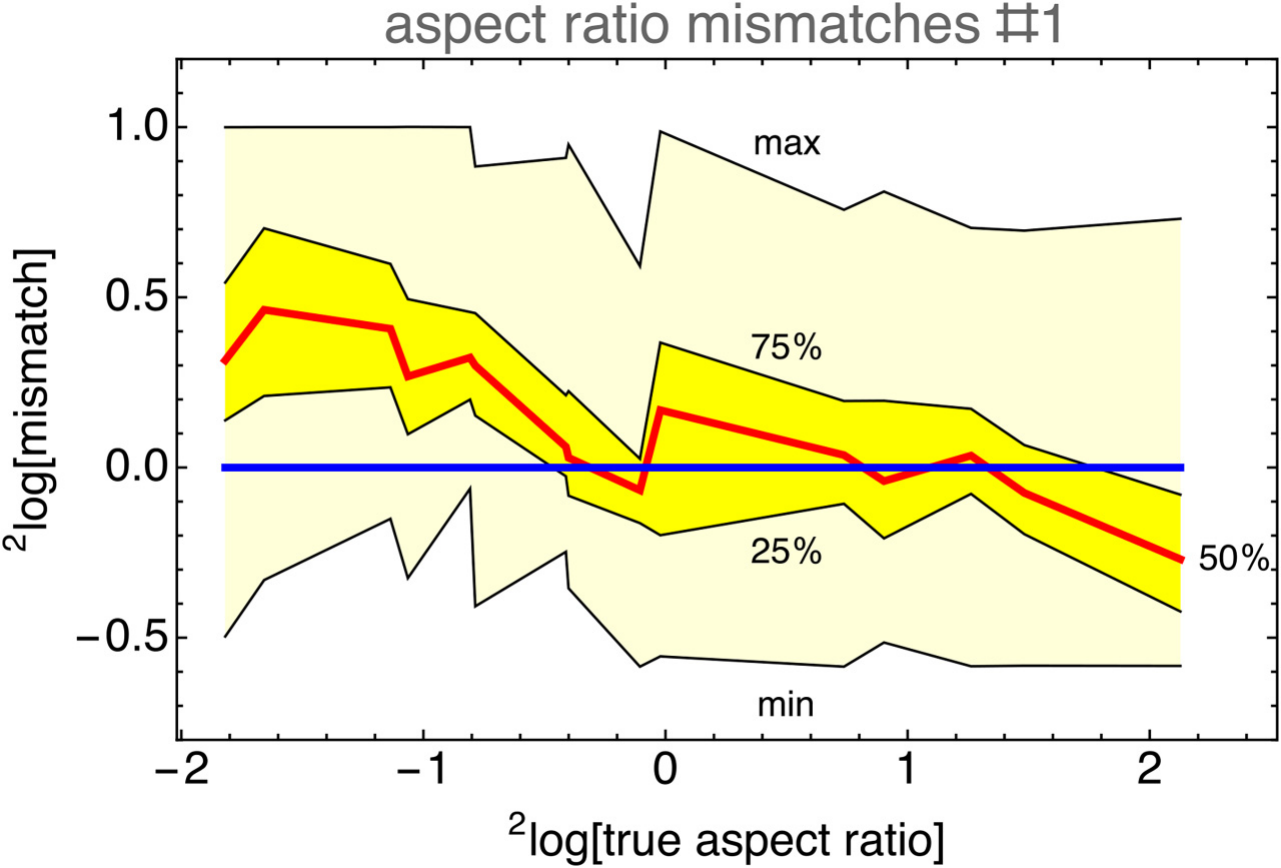
Range, interquartile range, and median as a function of true aspect ratio, computed over all participants. Notice the common trend toward some common preferences.

We also find the expected tendency toward some “neutral” aspect ratio (see Figure 34). All but one participant showed a slope in the same direction.

**Figure 35.**
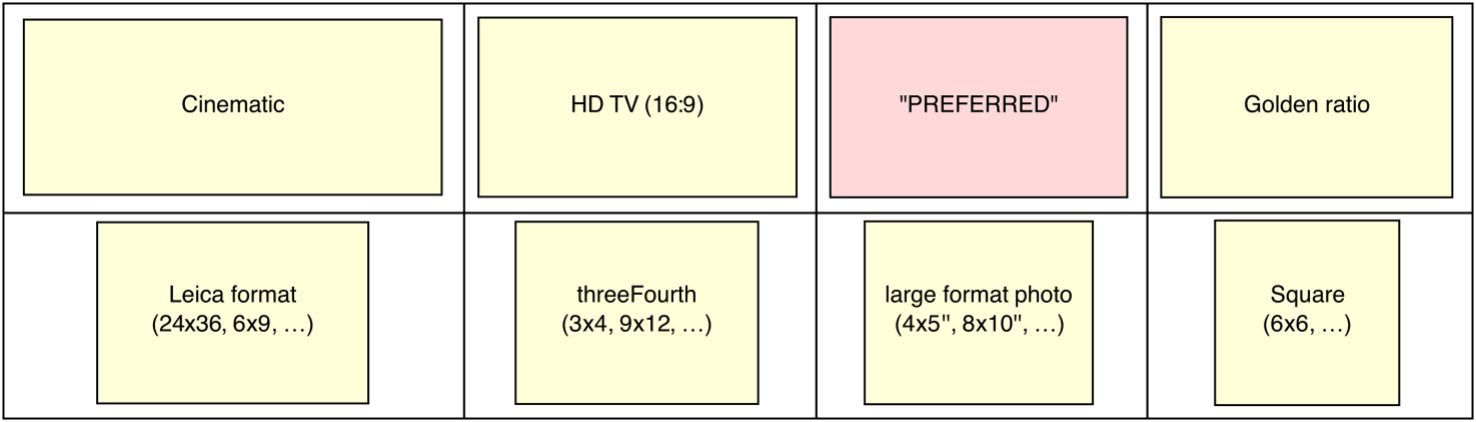
The overall preferred aspect ratio compared with a number of commonly encountered aspect ratios.

There is a wide range of “neutral” aspect ratios, the overall preference being about 5:3 ( 1.71 , see Figure 35). Of course, given the wide range in individual “neutral” values (see Figure 34), the overall value is very imprecise.

#### Epilog

All participants expect a concordance between pictorial content and aspect ratio. There is also evidence for a preferred or “normal” aspect ratio. Many artists will welcome the fact that this overall preferred aspect ratio is not that far from the classical “Golden Ratio” (Pacioli, 1509).^26^ In view of the spread over the group of participants one should not put too much importance on that.

## Mutual Comparisons

The general expectation is that larger numbers of observers and varieties of tasks should render it possible to detect inhomogeneities in the generic population and possible inconsistencies and mental fragmentations in aspects of pictorial awareness.

Of course, differences might well be due to random behavior, sloppiness, misunderstanding of the task, the use of various heuristics in reflective thought, and so forth. This is no doubt that all the above will occur to various degrees. It is not different in regular experiments, except that such differences are usually masked through the use of many repeats and the reporting of average results. The present experiment was expressly designed to reveal differences and in that respect, it can be reckoned successful.

How to decide on such issues as whether certain participants might have selective blindness to pictorial depth, or whether they respond by cognitive judgment instead of the intuitive imagination? There are no objective criteria. Different from classical psychophysics a “God’s Eye View” (Koenderink, 2014) is not available.^27^

The only way to proceed is by reasoning from the comparison of all aspects of responses over a variety of tasks. One criterion we use is based on the prior assumption that most—though by no means all—instances of a fairly well-defined generic population will be similar. After all, the participants are of the same biological genus (minor anatomical or physiological variation) and have similar (although not identical) cultural backgrounds. Thus, median responses over a variety of stimuli can (with care) be used as a common benchmark.^28^

Such a benchmark then takes the place of the actual (or “veridical”) stimulus parameters in classical psychophysics. In experimental phenomenology, there is no such thing as “veridicality.” If someone reports a pink elephant when looking at the *Gioconda* there is no way to consider him/her wrong. True enough! But this addresses an issue that should be faced squarely. For even if (we guess) most people would see a portrait of a woman (many art historians might “see” *Lisa di Antonmaria Gherardini di Montagliari*), none are in a position to judge a likeness. The similarity of their awareness would be limited to a generic “woman with such and so remarkable features.” If asked for the color of the eyes, most would probably need an additional look. What is noticed strongly depends upon the task.

Is anyone right here? It is probably best to go with the majority vote. After all, no one of us has to gain. This votes the pink elephant out. But can we say the singular person was wrong in experiencing that?

No way, how could we? There are no rational arguments to draw on. But one thing we can do is to combine the arguments we have. None of these may be totally convincing. But—as done in the courtroom—can we build a case? A sufficient number of unlikely coincidences soon becomes compelling. We feel we can build a case, at least in some instances. But none such a case can ever be totally conclusive. It can only be based on circumstantial evidence or indications. One should wield an academic restraint.

Anyway, we’re ready to go with rational arguments instead of “hard data,” there being no other option. In experimental phenomenology one cannot “let the data speak” as is often—right or wrong—done in the exact sciences.

### Comparison of Response Times

Most of the tasks are quite short:

- Visual field extent: This short task took one to two minutes.
- Box–shape in one-point perspective: This task took two to three minutes. Median response time per trial was 12 seconds, interquartile range of 10–18 seconds;
- Spatial attitude in pictorial space: Over all participants the setting took anything between half a minute and three minutes.
- Pictorial depth: This large task took 8 min as a median value, with an interquartile range of 6–11 min. A single trial takes 23 s (median) with an interquartile range of 17–30 s.
- “Presence” in pictorial scenes: This task took about 6 min (median) with an interquartile range of 5–8 min. This time is largely taken up by computer delays. A single trial took 18 s, interquartile range 13–23 s.
- Aspect ratio of pictures: This task took 4 min (median) with an interquartile range of 3–5 min; a single trial took 11 s (median) with an interquartile range of 8–16 s.

Additional time is spent on formal explanations of the task. Participants could take their time in reading the formal instructions. In practice the whole session (all six tasks) took less—sometimes much less—than an hour.

**Figure 36.**
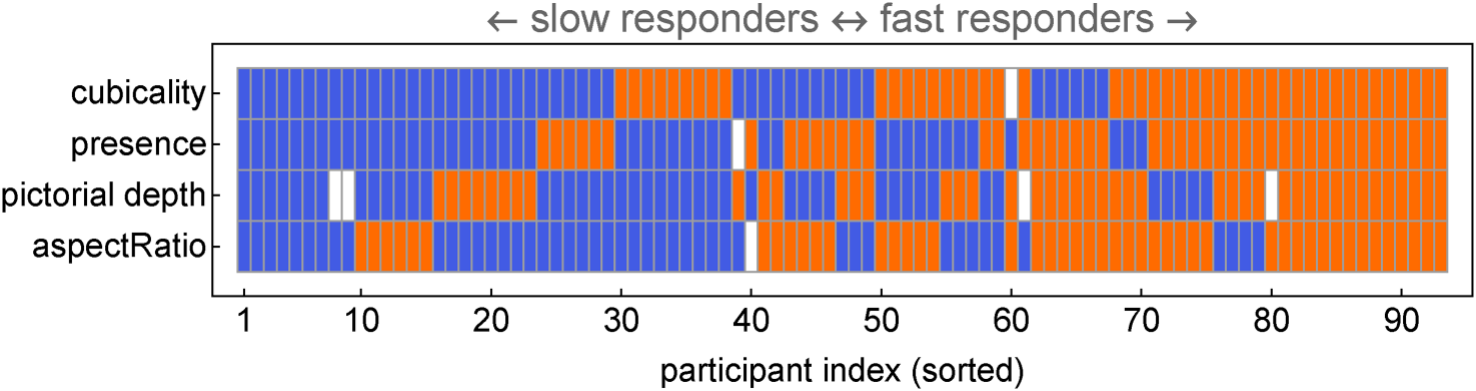
Fast and slow responders are defined by their relation to the median (per task). (We included only those tasks for which the response times make sense.) Blue is slow, orange is fast, and white is neutral (the median). The sorting order makes it easier to assess the distribution by eye. The z -score for most slow (at least three out of four) is 4.0 , and for most fast it is 2.5 . Apparently, participants who are slow or fast in one task tend to be slow or fast in other tasks.

There is a major variation in response times. As expected, a participant that is slow or fast in one task turns out to be often slow or fast in another task too (Figure 36).

It is hard to use such data. On the whole, fast is good because the participant is likely to act on the visual impression instead of a judgment in reflective thought. But a very fast observer may just be careless or sloppy. For instance, a very slow observer may be interesting because it might indicate some variety of dysfunctional stereopsis. But a very slow observer may well spend time in computations on some heuristic. Who knows?

One way is to compare response speed with settings. We find that participants who fail to correlate with the overall median overlap with the quartile of slowest responders.^29^ It seems likely that these people find themselves uneasy at the tasks, perhaps because it makes no intuitive sense to them.

### Comparison of Spatial Tasks

Theories of spatial tasks are often based on geometrical optics and geometry. In fact, many authors consider it to be the only rational approach. Berkeley’s (1709) powerful arguments against such an approach appear largely ignored or ridiculed:

But those lines and angles through which some men claim to explain the perception of distance are not themselves perceived at all, and by people unskilful in optics they‘re never even thought about. (A.12. at https://www.earlymoderntexts.com/assets/pdfs/berkeley1709.pdf)

Given this common base, one certainly expects to find relations between the extent of the visual field, the mismatches in settings of cube perspective, and the visual estimate of spatial attitude.

Thus, our set of experiments yields a welcome check on the validity of this almost universally adapted prior conviction. The result appears clearcut to us. Such an expectation is not born out at all.

The Kendall τ rank correlations of the visual field (vf), the visual estimate of spatial attitude (sa) and the mismatches in settings of cube perspective (cp) are:

vf–sa: Test statistic −0.013 , P value =.861 .
vf–cp: Test statistic +0.021 , P value =.779 .
sa–cp: Test statistic −0.100 , P value =.179 .

In no case can the null hypothesis that the populations are independent be rejected at the 5% level based on the Kendall τ test. The correlations are so small as to be of no interest at all.

The bottom line is that there are no empirical relations between the various spatial tasks. Participants act as if these tasks were completely unrelated. This seems to put Berkeley on the right.

In the past, we have developed and tested the tasks separately (vf: Koenderink et al. (2010b); sa: Koenderink et al. (2010a); cp: Pont et al. (2012)), but unfortunately there was no way to mutually correlate them. Most people in the field would probably expect such correlations to exist. One would expect them in the familiar Marrian framework, or in one of the models of the “inverse optics” variety. One way to hold on to such beliefs would be to consider the present results useless due to sloppy methods. That may be. But we feel this is a little too easy and look forward to better methods applied to similar group sizes.

## Conclusions and What Next?

In retrospect, we are happy with the six tasks. Participants enjoyed doing them and were not for a moment bored—as is all too common in more regular research. This is important to us, because it implies that the participants were fully involved with what they were doing, whatever that might be. It is not possible to know what people are doing, all one can do (after launching the task) is to record responses. These records suggest that most likely not all participants did the same thing. Of course, that is exactly what we are after in this study. Different from common practice, we treat these differences not as “noise,” but as *data*. In regular studies, one has rarely another option but to go for the noise option. Here we are indeed in a position to take the data seriously because of the unconventional design.

### Conclusions

#### Nature of psychogenesis

From an a priori, conceptual viewpoint, one describes all spatial tasks in terms of geometrical optics and geometry (Pirenne, 1970; Cooper et al., 2012). That leads to the prediction that the responses for such tasks are likely to be related, because they imply the use of shared algorithms. Given that such relations are empirically nonexistent, this a priori notion should be dropped.

Participants act as if they used mutually independent heuristics for the various tasks. We do not speculate on whether such heuristics are applied in psychogenesis (pre- or proto-awareness) or in reflective thought.

That an application of mutually independent heuristics is not such a strange notion becomes clear when one changes the a priori viewpoint to a proper biological one (Uexküll, 1921, 1934, 1936). Then tasks are likely to be handled according to present goals and situational awareness. From ethology, we know that this is the way biological organisms tend to solve their problems. This would also explain why the results of different tasks may appear to lead to mutually inconsistent results.

Such inconsistencies derive from an unsound conceptual viewpoint. From the biological viewpoint, there are no inconsistencies, only responses to different tasks.

This is also found in the human belief system. It explains the many inconsistencies found in practice. They derive from the mistaken assumption that humans are *rational agents*. But humans are *biological agents* that act according to circumstances and goals (Hoffman, 2009; Koenderink, 2019a, 2019b, 2019c). We are not different from our fellow animals in this respect.

#### Variations over the population

Are there interobserver differences in the ability to wield monocular stereopsis? There are reasons to believe so (Koenderink, 2015b).

Reasons derived from major deviations from the median population response. If a participant fails to correlate with median pictorial depth over a fair range of pictures, then its monocular stereopsis is definitely atypical. Since there isn’t much room for major variation^30^ one concludes that the monocular stereopsis is absent or weak. Many of such participants did not see Lord Kitchener’s finger stick out of the picture plane. That would indeed make sense when stereopsis was lacking.

We read this as an indication that something like a quarter of the population might lack monocular stereopsis in the strict sense: a quality of pictorial awareness. This agrees with numerous informal and formal facts we collected over several decades. Remember that it is not obvious how to spot this in regular interactions.^31^ An inquiry by way of Socratic questioning^32^ yields mainly examples of fragmentary and inconsistent belief systems.

There may well be gradations in the ability of monocular stereopsis, we have no way to know. Artists might say that some customers of galleries “look with their ears,” and know that it often takes artists themselves years to gain the knack “to see.”

A lack of monocular stereopsis is perhaps to be read as an indication of *aphantasia*, or “mind blindness” (Galton, 1880; Zeman et al., 2015), or perhaps a lack of the ability of *perspective taking* Piaget (1977).^33^ It might also be read as weak creative imagery.^34^ It is clear to us that all participants know quite well how to look at and work with pictures on a daily basis. The literature about imagination and aphantasia largely deals with “visions” in the absence of pictures. “Perspective taking” in the optical sense is most often understood as being able to visualize a pictorial scene as seen by another person. All that is irrelevant to the present issue. The crucial ability is the ability to experience the *Fernbild*^35^ in the presence of a picture. The “perspective taking” involves putting the mental eye at infinity (Koenderink & van Doorn, 2023), not at another person’s vantage point. Even in Hildebrand’s time (his book dates from 1893) only one art historian (Heinrich Wölfflin (1915)) and few scientists understood him. This has hardly changed throughout the years. It is to be deplored, because Hildebrand—as an artist—wrote an important work on the theoretical phenomenology of iconic perception. Up to today, it remains the definitive reference.

#### Artistic praxis and “official perspective”

Ever since the description of the correct method of depiction (*De Pictura* of 1435) by Leon Battista Alberti (1435), artists have systematically broken the official rules. The reason is simply that they were in the business of making depictions “look good,” whereas the correct method often made them “look wrong.” There is much literature on the nature of such “perspective deformations” (Pirenne, 1970), but preciously little data. The reason is probably that conventional methods of experimental psychology do not let one address the relevant issues. “How things look” is a matter of experimental phenomenology (Albertazzi, 2013). The present findings indeed throw additional light on the topic.

Relevant instances are

- In pictorial vision, the divergence of the actual visual rays (Euclid, 1943, orig. ca.300bce) is ignored. The picture is experienced as a view from infinity, the “mental rays” are mutually parallel. The physical representative of a mental ray is a “pixel” (point of the picture plane) augmented with a virtual depth dimension. The depth is one-dimensional (a “line”), but is purely virtual and has no relation to the physical eye (Koenderink & van Doorn, 2023). It is why the vantage point of the viewer is largely irrelevant in pictorial vision and the conventional advice to put the eye at the right perspective center is ineffective. The artists handle this by “counter-rotating” eccentric objects. This prevents a frontal attitude from appearing as a (partial) profile. It is almost universally applied, but rarely noticed because it *looks* correct instead of odd.
  This is indicated by the results from the box-shape and spatial attitude tasks.
- Spatial attitudes of objects near the left and right frame borders have to be “corrected.” True perspective relates them to the pyramid of diverging visual rays, but pictorial perception relates them to the mutually parallel mental rays. In case the angular difference is about 30∘ or more, it is easily apparent. For instance, an eccentric sphere is depicted with a circular instead of an elliptical outline. This is “wrong” according to Albertian perspective rules, but it *looks* right.
  This is indicated by the results from the spatial attitude task. It is a strong effect that gave rise to much—still unresolved—discussion in the literature (Pirenne, 1970).
- The size of a picture is—as far as perspectival effects go—largely irrelevant. A “visually correct” depiction of a cube in a one-point perspective does not change between an apparent shallow slab and a deep corridor. Instead, it keeps looking like a cube. This is also why you cannot obtain a “normal view” of a wide-angle photograph by viewing it from very close, or of a shot taken with a telelens by looking from afar. The former always appears in “accelerated perspective,” whereas the latter always appears “compressed.” Artists know this and use this, which is why one rarely sees “wide angle distortions” (common enough in photography) in paintings.^36^ Again, it is necessary to depict “wrong” in order to make things *look* right.
  This is indicated by the result of the spatial attitude task.

Of course, hard-core scientists who base their expectations on the (usually tacit) assumption that psychogenesis runs geometrical algorithms that implement “inverse optics” (Marr, 1982; Poggio et al., 1985; Mallot et al., 1991; Pizlo, 2001) will retort that the present “explanation” does not explain anything at all, because it is a mere description of visual experience.

We grant that. However, we feel that a valid description of visual experience (phenomenology) is preferred over a principled theory that fails to account for the phenomenal facts.

The relevant approach is theoretical phenomenology. There is no reason why that could not be formal and precise (Koenderink & van Doorn, 2023). Moreover, fitting the facts is the primary concern, sophisticated mathematics is—or at least should be—secondary.

### What Next?

Our set of tasks turns out to lead to quite informative results, with the possible exception of the “presence” task. They yield mutually complementary data in a relatively short session. Most participants enjoyed taking part in the project. They come up with more questions than we are able to answer. This is important, because it suggests that we succeeded in avoiding artificial, laboratory settings.

What is striking is that we are dealing with really huge deviations from “veridical” expectations and that interobserver differences are likewise appreciable. Although too large to ignore, much of this type of variation has never been properly mapped out.

The present approach effectively enlarges the scope of empirical phenomenology. There are numerous aspects of the vision for which the distribution of the population remains effectively unknown. Moreover, it is virtually impossible to foresee the full range of heuristics observers might deploy.

## Notes

1. In phenomenology one often uses “presentation” instead of “intuition,” but it is really the same thing. “Presentations” simply appear as awareness and do not involve (as “representations” do) reflective thought, involving cognitive “judgments.”

2. The topic may be purely imaginary, say God on his throne. In that case, there is no physical scene.

3. What is an “iconic object?” Artists think of it as a “unity” in the sense of a “single thought,” where the “thinking” is of a sentient rather than sapient nature Baumgarten (1735). For vision science, the best definition might be a “Gestalt.” Anything that contains unnecessary parts thereby is not iconic because it necessarily involves reflective thought. A Gestalt may appear in pure awareness and does not (necessarily) involve reflective thought. Pure awareness is a “here and now” (or “actuality”) that has no proper parts, although it may be complex.

4. In the pictorial arts the chronotope (unity of space and time (Bakhtin, 1981)) is provided by the artist. In cubism, the task of arriving at such a unity is shifted to the spectator. In each case, the chronotope is a mental achievement.

5. With “iconicity,” we refer to immediate visual awareness, in the absence of reflective thought. From this view “cognitive art” is not necessarily “iconic,” although it might be. A face seen in a cloud is iconic in the sense that it hardly can be a conceptual judgment.

6. Here we evidently do not mean mere conventional signs, but rather such things often denoted “cues” in vision research. In ethology, Jakob von Uexküll uses “Merkmale” and “Wirkmale” (Uexküll, 1934, 1936). This fits the understanding of artists who see the Merkmale (or “cues”) and paint Wirkmale (actions that will—perhaps covertly—affect future viewers).

7. This is a matter of definition. Think of the adaptive coloration of animals and plants. However, intentional picture crafting is rare in the animal kingdom.

8. Neither extreme sentience, nor extreme sapience recognize either jokes or art. Explaining a joke kills it, so does explaining (visual) art. Visual art aims at the eye, and conceptual art aims at the ratio. Whereas words kill visual art, they assist conceptual art. In effect, the right words may render the actual conceptual art work superfluous. In contradistinction, visual art can do without titles. Titles are often supplied by gallery owners rather than the artists.

9. As commonly used in music, pictorial art, cinema, writing, and so forth. In order to be successful a painting should not only attract the eye, but also “hold” it.

10. A wisdom from the artist is that variations, steps, and so on, should come at least in groups of three and at most in groups of seven. This has to do with catching and capturing attention.

11. Virtually all pictorial artists consider that nonsense. They are right because the disparity field of a picture only serves to indicate its flatness. But even in daily vision binocular disparity is highly overvalued. As much as a third of the population misses it to some extent (Richards, 1970) and is not even aware of that.

12. “Strong points,” and so forth (Dunn, 1995; Hamm, 1972; Hanks & Belliston, 1992). The frame forces a closed composition and defines the frontal (“Alberti’s”) plane. In pictorial perception “depth” tends to depart from the frontal plane (Hildebrand, 1893).

13. Both public domain.

14. Mathematica is available on many platforms.

15. Most artists would rather close an eye.

16. The formal instruction sheets are available on the publisher’s website.

17. Monitored by counting certain key presses, not counting key-repeats.

18. We informed them that estimates ranging from 0∘ – 360∘ are to be expected in the normal population.

19. *Habitus* is used as “the characteristic form or morphology of a species” in botany, “the characteristic external shape of an individual crystal” in mineralogy, and so forth.

20. This is the distinction between *Wirklichkeit* and *Realität*, which is hard to express in English. Here we use “actuality” for *Wirklichkeit* and “physical reality” for *Realität*.

21. It is the distinction between *Bild* and *Vorstellung*, which is hard to express in English. We use “picture” for *Bild* and “image” (an “intuition”) for *Vorstellung*.

22. The glaring inconsistency is that you point from visual space into pictorial space, two ontologically distinct realms (Koenderink, 2015a). Is such possible at all? Frankly, it is a step in the dark. As it turns out to be the case, most participants readily do the task and even feel it to be “natural.”

23. “Lord Kitchener,” a 1914 advertisement by Alfred Leete.

24. Note that the case of Figure 30 top is special because the interior architecture contains no rectilinear horizontals.

25. DIN A-series, cutting a piece in half yields pieces of the same aspect ratio.

26. The “Golden Ratio” is ( $(1+\sqrt{5})\;:\;2$ . The rational approximation 5:3 is about 3% too high.

27. In classical psychophysics, a physical measure tends to suffice as a God’s Eye View. In terms of classical—before the quantum revolution—physics that makes sense.

28. When there are indications of distinct subpopulations a cluster analysis might serve to apply this heuristic cluster-wise. This necessarily involves very large groups and, thus is unlikely to be of use in the present case.

29. Using the Jaccard index (Jaccard, 1912) at the 5% level.

30. It is unlikely to consistently see backgrounds in front of foregrounds, for instance.

31. Again, this group will still see the man in front of the woman in Figure 24.

32. As tried in postsession talks.

33. Of course, these terms are to be taken in an “optical,” rather than social, or emphatic sense. Pictorial artists talk of “being able to see,” or of customers who “see with their ears.” A lack of “The Eye” by no means implies walking into doors. The eye doctor will find no abnormal condition. Thus, vision science has no clue as to what the artists might mean.

34. Perhaps Goethe’s “exakte sinnliche Phantasie” (Hennigfeld, 2021) or Baumgarten’s (1735) definition of æsthetics as the “Science of Sensible Knowledge.”

35. A truly iconic entity, in the sense of Adolf Hildebrand (1893).

36. Corrections are largely absent in early renaissance painting and not infrequently in late nineteenth-century work after the audience got used to photographic renderings.
